# Neoproterozoic rifting in the Upper Yangtze Continental Block: Constraints from granites in the Well W117 borehole, South China

**DOI:** 10.1038/s41598-017-12764-y

**Published:** 2017-10-02

**Authors:** Deng-Fa He, Di Li, Chuan-Xin Li, Ying-Qiang Li, Qing-Hua Mei

**Affiliations:** The Key Laboratory of Marine Reservoir Evolution and Hydrocarbon Accumulation Mechanism, The Ministry of Education, China University of Geosciences, Beijing, 10083 China

## Abstract

Well W117 in the Sichuan Basin reveals a suite of ~814 Ma quartz monzonites, unconformably overlain by Sinian clastic and carbonate sediments. The quartz monzonites contain no muscovite and amphibole, and are characterized by high SiO_2_ (72.26–77.93%), total alkali, and TFe_2_O_3_/MgO content, and low P_2_O_5_ and CaO abundance, with variable A/CNK ratio (0.93–1.19), classified as metaluminous to weakly aluminous highly fractionated I-type granites. They are preserved in the Neoproterozoic rift and exhibit restricted negative ε_Nd_(t) values (−7.0 to −5.2) and variable zircon ε_Hf_(t) values (−13.9 to 2.3), suggesting their generation via melting of both ancient and juvenile crustal materials in an extensional setting. Their parent magmas were formed in a low-temperature condition (831–650 °C) and finally emplaced at ca. 9–10 km below the surface, indicating that the intrusion underwent exhumation before the deposition of Sinian sag basin. Such geological processes, together with evidence for Neoproterozoic structures in the surrounding area, support that the Upper Yangtze craton experienced two main phases of rifting from 830–635 Ma. The Well W117 granites and its overlying sediments record a geodynamic evolution from orogenic collapse to continental rifting, and to thermal subsidence, probably related to the Rodinia supercontinent breakup.

## Introduction

The Neoproterozoic was a critical period in the tectonic evolution of the South China Block^1–5^, as evidenced by the extensive occurrence of mid-Neoproterozoic bimodal plutonic and volcanic rocks throughout the Upper Yangtze continental block^2,6^, with two prominent age peaks, at ~820 Ma and ~750 Ma^2,4,7^. These mafic and felsic magmas, with variable isotopic signatures, reflect the generation of new juvenile crust and reworking of ancient crust in this interval. It is widely believed that their formation was associated with the evolution of the Rodinia supercontinent^2,3,8–11^. In the past decades, a large number of ca. 860–750 Ma rift-related basaltic magmas^12–20^ and well-preserved Neoproterozoic rift sequences^21–23^ were recognized along the margins of the Upper Yangtze continental block, implying that the rifting played an important role in the Neoproterozoic evolution history. However, its geodynamic mechanism is still hotly debated. Some scholars consider the Neoproterozoic rifting events as a result of slab subduction based on the development of arc-type magmas along the northern and western margins of the Upper Yangtze continental block^10,24,25^, while others suggest that those near the Jiangnan Orogenic Belt may be related to orogenic collapse^4,26^. Li *et al*. (2003), taking into account the petrogenesis and distribution of Neoproterozoic magmas, correlated the rifting around the margins of the Upper Yangtze continental block with the episodic activities of the mantle plume or superplume beneath the Rodinia supercontinent^2^. This viewpoint is also supported by high-temperature komatiitic lava^27–29^, OIB-type alkaline basalts^30,31^ and continental flood basalts^30,32,33^. Such competing interpretations need to be explored for further understanding the geological architecture and evolution of the Upper Yangtze continental block and its position in the Rodinia supercontinent^1,34^. Recent geophysical data clearly show the continental structure of the Upper Yangtze block with a series of Neoproterozoic faulted structures and minor Proterozoic gabbroic intrusion preserved in the cratonic basement^35^. However, little is known about the filling sequence and tectonic evolution of these Neoproterozoic faulted basins due to the widespread late Neoproterozoic to Cenozoic sedimentary cover (e.g., the Sichuan Basin). In the course of ongoing hydrocarbon exploration in the deep Sichuan Basin, a few wells have drilled into Neoproterozoic strata and their associated intrusions. These valuable borehole data (both well-logs and core samples), can undoubtedly provide us with a better understanding of the Neoproterozoic rifting of the Upper Yangtze continental block.

In this study, we report a Neoproterozoic stratigraphic succession and associated granitic pluton from Well W117, a borehole drilled into the Weiyuan High in the Sichuan Basin. We present geochronological, geochemical, whole rock Sr–Nd isotope and zircon Lu–Hf isotope results from the Neoproterozoic granites, and make a detailed study for their source, petrogenesis and P-T conditions. Our aims are to reveal the formation, emplacement and exhumation histories of the Well W117 granites, to better understand the Neoproterozoic rifting processes of the Upper Yangtze continental block and discuss their tectonic implication for possible driving mechanism.

## Geologic background and sampling program

The Upper Yangtze continental block is an important component of the South China Block, bounded to the west by the eastern Tibetan Plateau, to the north by the Qinling-Dabie Orogenic Belt, and to the east by the Jiangnan Orogenic Belt. It is widely considered that its final collision with the Cathaysia continental block occurred in the early Neoproterozoic (ca. 900–880 Ma)^1,2,13,21^. The Upper Yangtze Block is composed of Archean to Early Neoproterozoic basement complexes containing sandy to argillaceous metasedimentary strata^36,37^, overlain by a thick late Neoproterozoic to Cenozoic sedimentary successions^38^. Zircon grains from felsic granulite xenoliths in the Upper Yangtze continental block suggest that Archean crust is widespread at middle to lower crust level^35^, despite its surface exposure being limited to a few locations such as the Kongling TTG complex (2.9–3.3 Ga)^36,39^. While several Paleoproterozoic (1.97–2.03 Ga) tectono-thermal events are recorded in the northern part of the craton^40,41^, Paleo- to Mesoproterozoic (1.7–1.0 Ga) sedimentation in the southwestern Upper Yangtze continental block was mainly associated with magmatic activity^42–44^. Early Neoproterozoic strata, metamorphosed to the greenschist facies, are distributed continuously along the periphery of the Upper Yangtze continental block^39,45^, intruded by middle to late Neoproterozoic granitoids^2^. These metasediments and granitoids are unconformably overlain by a late Neoproterozoic (Sinian) to Middle Triassic marine sedimentary successions^21,46,47^.

**Figure 1:**
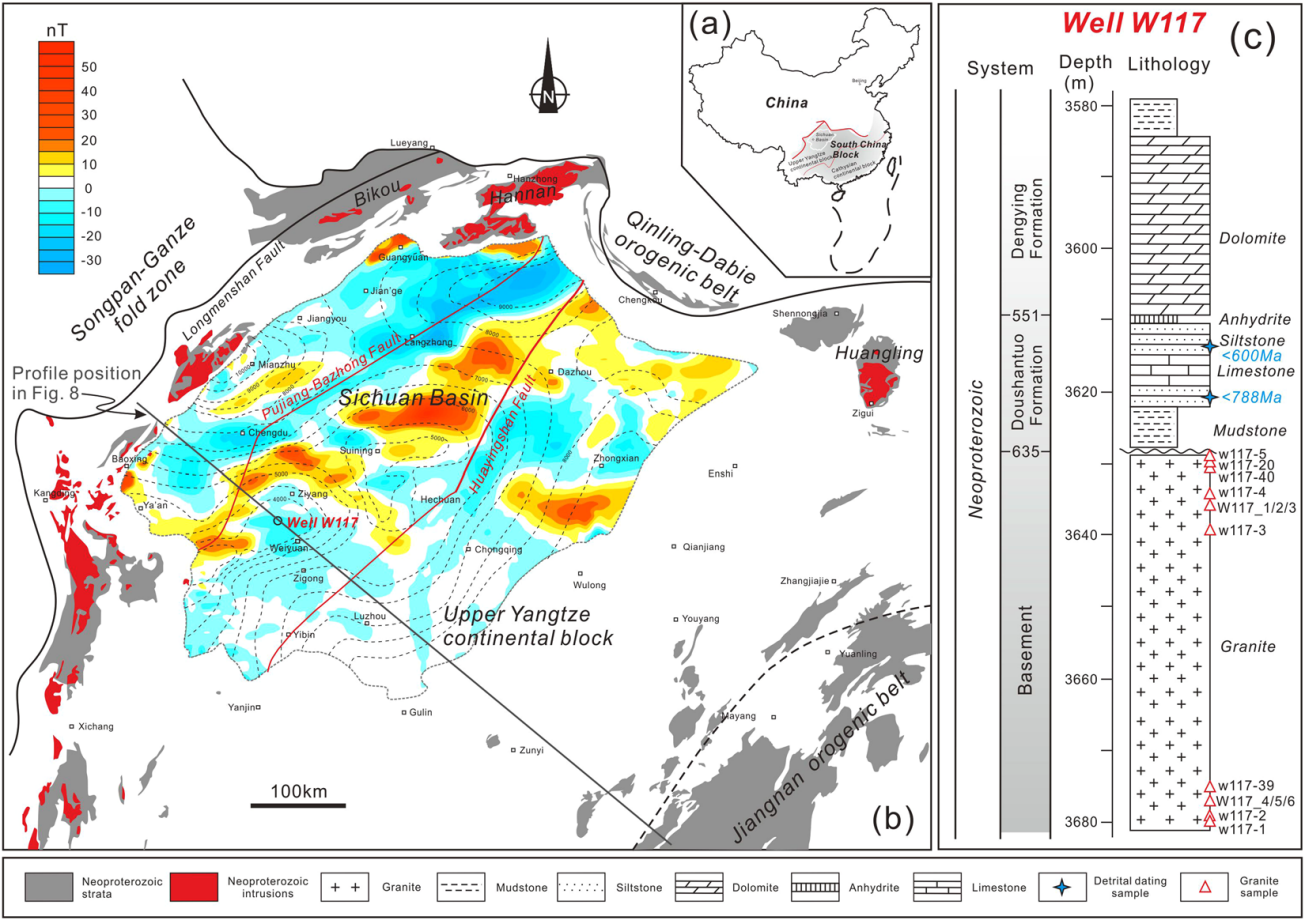
(**a**) Location of the Upper Yangtze continental block in South China. (**b**) Simplified geological map showing the tectonic relationship between the Upper Yangtze continental block and its adjacent tectonic domains, as well as aeromagnetic pole anomaly characteristics of the Sichuan Basin. (**c**) Neoproterozoic stratigraphic successions in the basin basement, as seen in Well W117. This figure is generated by Di Li, using CorelDRAW X6 created by the CorelDRAW Team under an open license (http://www.coreldraw.com/cn/product/graphic-design-software/).

The Sichuan Basin occupies the central portion of the Upper Yangtze continental block, and is one of the key areas for reconstructing its evolutionary history (Fig. 1a). Aeromagnetic pole anomaly data (PetroChina Southwest Oil & Gasfield Company (PCSOGC), 1990) suggest that the basin basement displays significant structure relief, with a ‘low–high–low’ pattern from east to west (Fig. 1b). Based on this apparent basement structure, the Sichuan Basin can be divided into eastern, central, and western tectonic units, bounded by the deep, NE-trending Huayingshan and Pujiang–Bazhong faults. The Weiyuan High exhibits a negative aeromagnetic anomaly, within the positive anomaly zone of the central Sichuan Basin. Borehole data from Well W117 (PCSOGC, 1985) reveal that this negative anomaly area in the basin basement represents a set of Neoproterozoic granitic pluton, unconformably overlain by the late Neoproterozoic Doushantuo and Dengying Formations (Fig. 1c). The Doushantuo Formation comprises mudstone, limestone, siltstone and minor anhydrite. The youngest detrital zircon U–Pb ages from two siltstone samples indicate that deposition did not occur earlier than 788 Ma (our unpublished data) (Fig. 1c).

**Figure 2:**
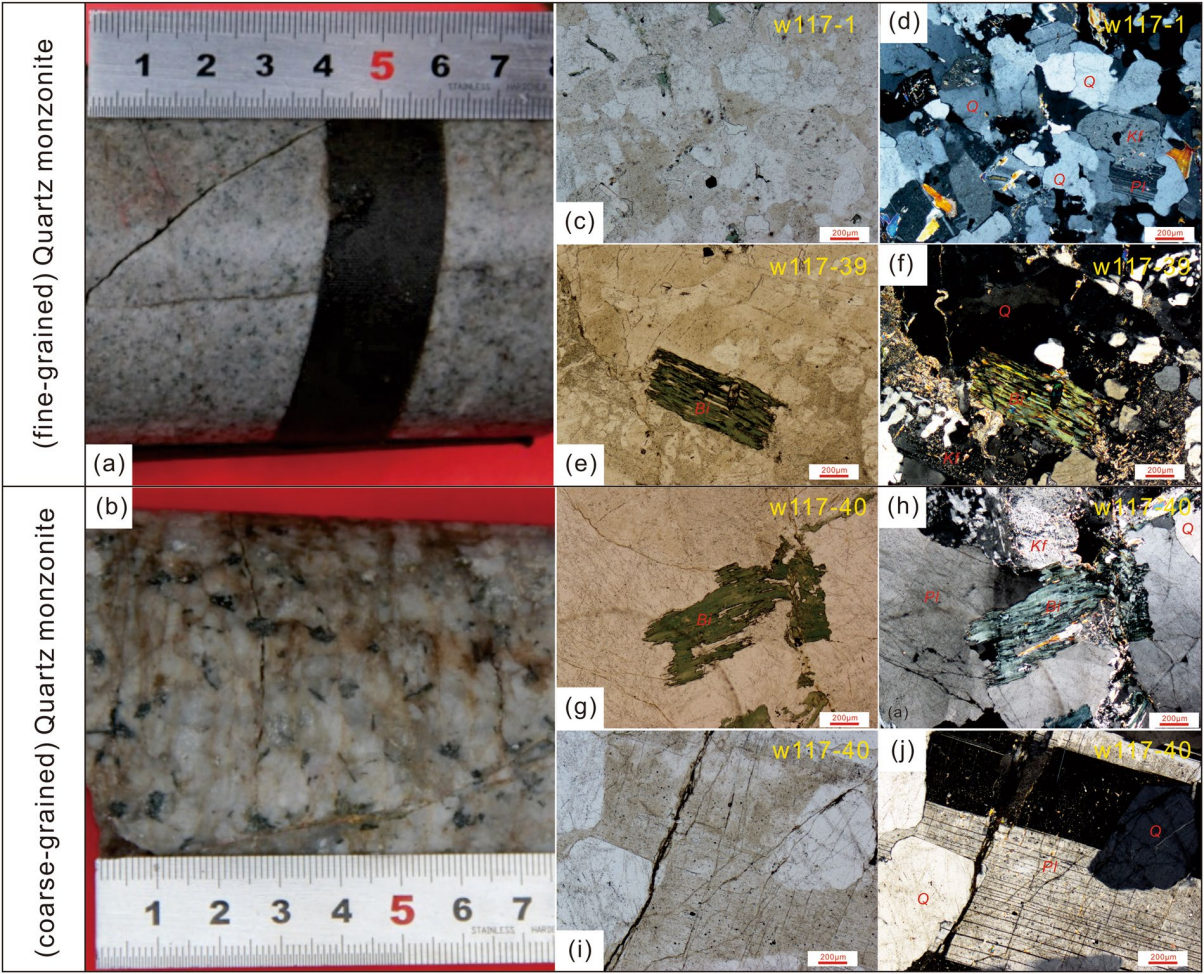
Photomicrographs (crossed nicols) and petrological characteristics of the Neoproterozoic fine- and coarse-grained quartz monzonites from Well W117 in the Sichuan Basin. Pl–plagioclase, Kf–K-feldspar, Bi–biotite and Q–quartz.

The granites making up the underlying basement can be divided into two groups based on their textural characteristics. The pluton forming the upper part of the drilled basement of Well W117 is composed of coarse-grained quartz monzonite, while the lower part of the basement is fine-grained (Fig. 2a,b). The fine-grained quartz monzonite shows porphyritic textures, and consists mainly of K-feldspar (35–40%), plagioclase (30–35%), quartz (~10%), biotite (5–10%), and minor accessory minerals. These rocks have a weakly schistose fabric, with biotite-rich zones aligned between feldspar- and quartz-rich areas (Fig. 2c,d,e and f). The coarse-grained quartz monzonite is pink in color (Fig. 2f) and is composed of K-feldspar (45–50%), plagioclase (30–35%), quartz (5–10%), with minor biotite, zircon, apatite and opaque Fe-Ti oxides (Fig. 2g,h,I and j). The quartz (0.6–1 mm) is xenomorphic, mostly filling gaps between coarse feldspar crystals that are 4–8 mm in size. Some minor alteration has occurred on the surfaces of feldspar crystals, forming sericite. Eight granite samples (four fine-grained quartz monzonites and four coarse-grained ones) were collected from Well W117 at depths of 3676–3679 m and 3631–3635 m. These samples are 3 cm × 6 cm × 9 cm in size and were processed for geochronological, geochemical and isotopic analysis, to reveal their formation age, sources, petrogenetic history and emplacement conditions.

## Results

### Geochronology of the quartz monzonites

**Figure 3:**
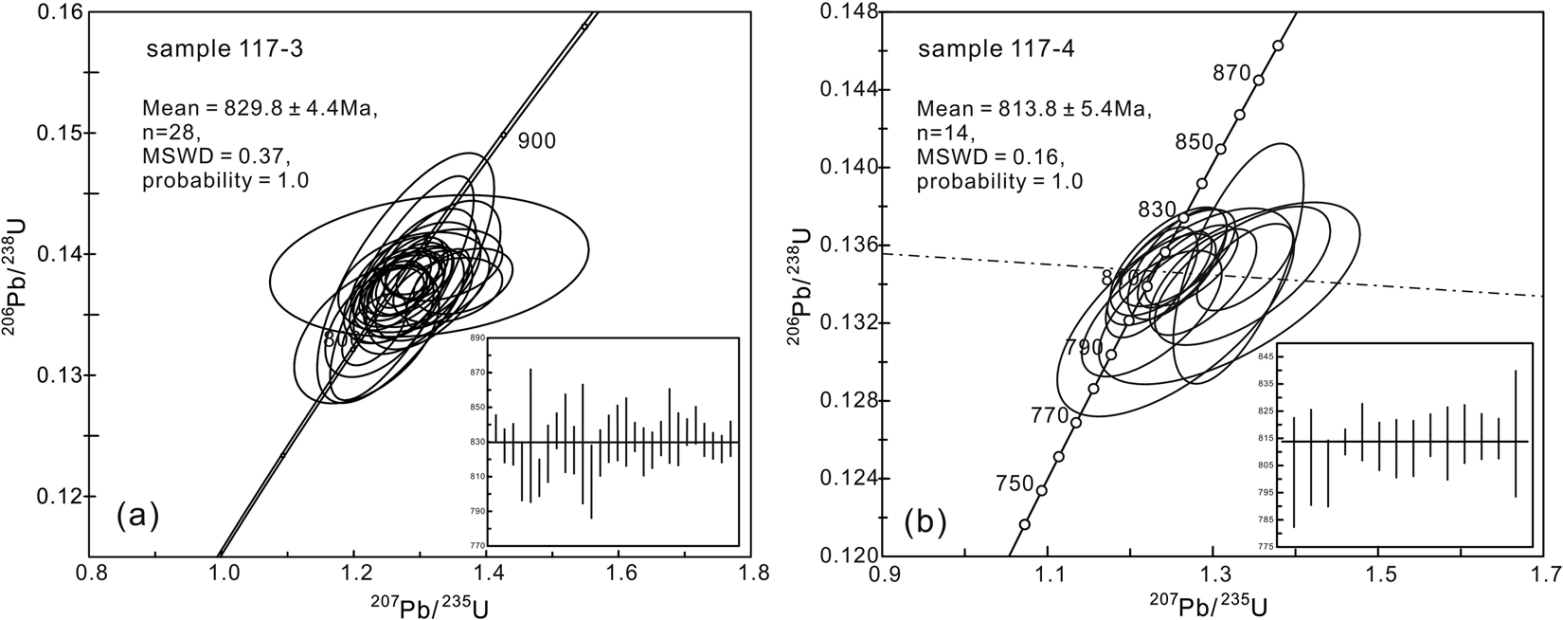
Zircon LA-ICP-MS U–Pb concordia diagrams for the (**a**) fine-grained (sample 117-3) and (**b**) coarse-grained (sample 117-4) quartz monzonites from Well W117.

The fine- and coarse-grained quartz monzonites (117-3 and 117-4) at the same depth (~3635 m) were selected for U–Pb dating by laser-ablation inductively coupled plasma mass spectroscopy (LA-ICP-MS) (Table 1S). Zircon grains from the samples are transparent and prismatic, with length/width ratios of 1 to 4. The cathodoluminescence (CL) images show that all of the grains have well-developed oscillatory zoning and lack visible inherited cores (Fig. 1S). They have high Th/U ratios (0.37–2.16), which, together with the good oscillatory zoning, indicate an igneous origin. Twenty-eight zircons from the fine-grained sample (117-3) yield concordant ^206^Pb/^238^U ages, with a mean of 829.8 ± 4.4 Ma (Fig. 3a), and fourteen analyses from the coarse-grained sample (117-4) yield an age cluster at 813.8 ± 5.4 Ma (Fig. 3b). These results constrain the formation age of the Well W117 quartz monzonites to ca. 814 Ma, in the mid-Neoproterozoic. The ca. 830 Ma zircons may be captured from wall rocks during the magma ascent.

### Major and trace elements

**Figure 4:**
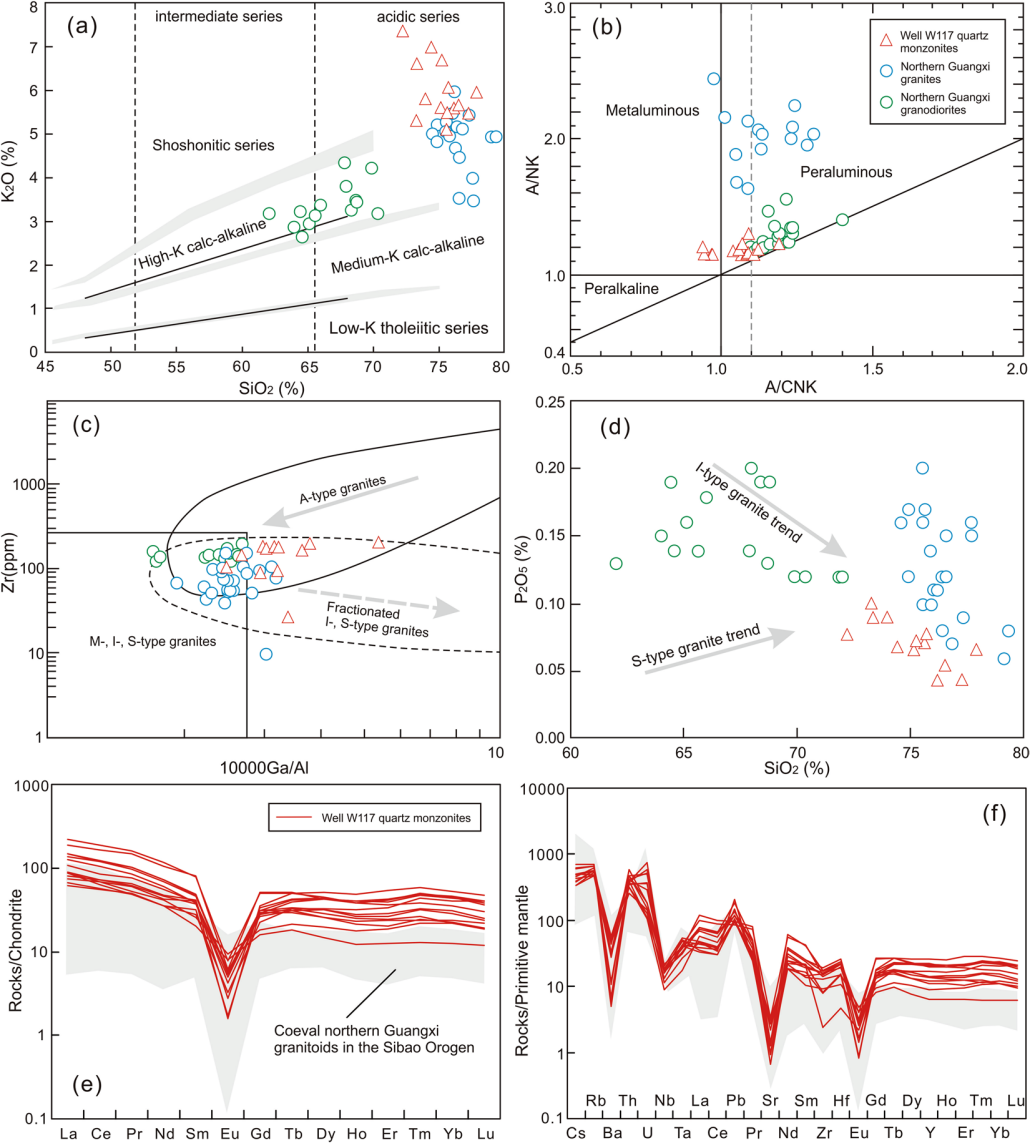
Geochemical diagrams for the Well W117 quartz monzonites: (**a**) K_2_O versus SiO_2_ diagram; (**b**) A/NK versus A/CNK diagram^73^; (**c**) Zr versus 10000 Ga/Al diagram^74^; (**d**) P_2_O_5_ versus SiO_2_ diagram for granites; (**e**) Chondrite normalized REE patterns; and (**f**) Primitive mantle normalized trace element spider diagrams. Chondrite and primitive mantle normalized values based on Sun and McDonough^75^. Data for the coeval Northern Guangxi granitoids in the eastern part of the Jiangnan Orogenic Belt are from Li *et al*.^2^ and Yao *et al*.^17^.

All major and trace element data from the Well W117 quartz monzonites, including previously reported data^48^, are listed in Table 2S. The quartz monzonites display a narrow range in SiO_2_ content (72.26–77.93%), although fine-grained quartz monzonites possess slightly higher abundances of SiO_2_ than coarse-grained ones (Table 2S). All are characterized by high total alkali content (K_2_O + Na_2_O = 7.52–9.58%), and they mostly plot within the shoshonitic series field on the K_2_O–SiO_2_ diagram (Fig. 4a). The moderate A/NK (1.12–1.30) and varied A/CNK (0.93–1.19) ratios indicate that these granitoids are metaluminous to slightly aluminous (Fig. 4b); that is consistent with the Barth mesonormative calculation result, which suggests that most of the samples should contain some biotite (1.83–5.11%), muscovite (1.40–6.78%) or minor amphibole (~5.4%). The quartz monzonites have variable 10000 Ga/Al ratios (2.47–5.37, Fig. 4c), and low MgO (0.16–0.82%), TFe_2_O_3_ (1.56–3.00%) and P_2_O_5_ (0.04–0.11%, Fig. 4d) contents.

Overall, the quartz monzonites from Well W117 are characterized by low total REE abundance (150–441 ppm) and coherent V-type chondrite-normalized REE patterns (Fig. 4e), with a relatively flat HREE distribution (Ga_N_/Yb_N_ = 0.55–1.44), and a pronounced negative Eu anomaly (Eu/Eu* = 0.04–0.44), as well as the clear “tetrad effect”. Notably, the coarse-grained quartz monzonites are more highly enriched in LREEs (La_N_/Yb_N_ = 3.97–7.04) than those of the fine-grained ones (La_N_/Yb_N_ = 1.36–4.91) (Fig. 4e). On the primitive mantle normalized diagram (Fig. 4f), they display enrichment in LILEs (e.g., Cs, Rb, Th and U), LREEs (e.g., La and Ce) and Pb, with depletion in Nb, Ta, Sr, and especially Ba relative to Rb and Th. These features match with those seen in the ca. 820–810 Ma granitoids in the Sibao Orogen^2,17^, similar to those of the post-collisional granites worldwide^49^.

### Whole rock Sr–Nd isotopes and *in situ* zircon Lu–Hf isotopes

**Figure 5:**
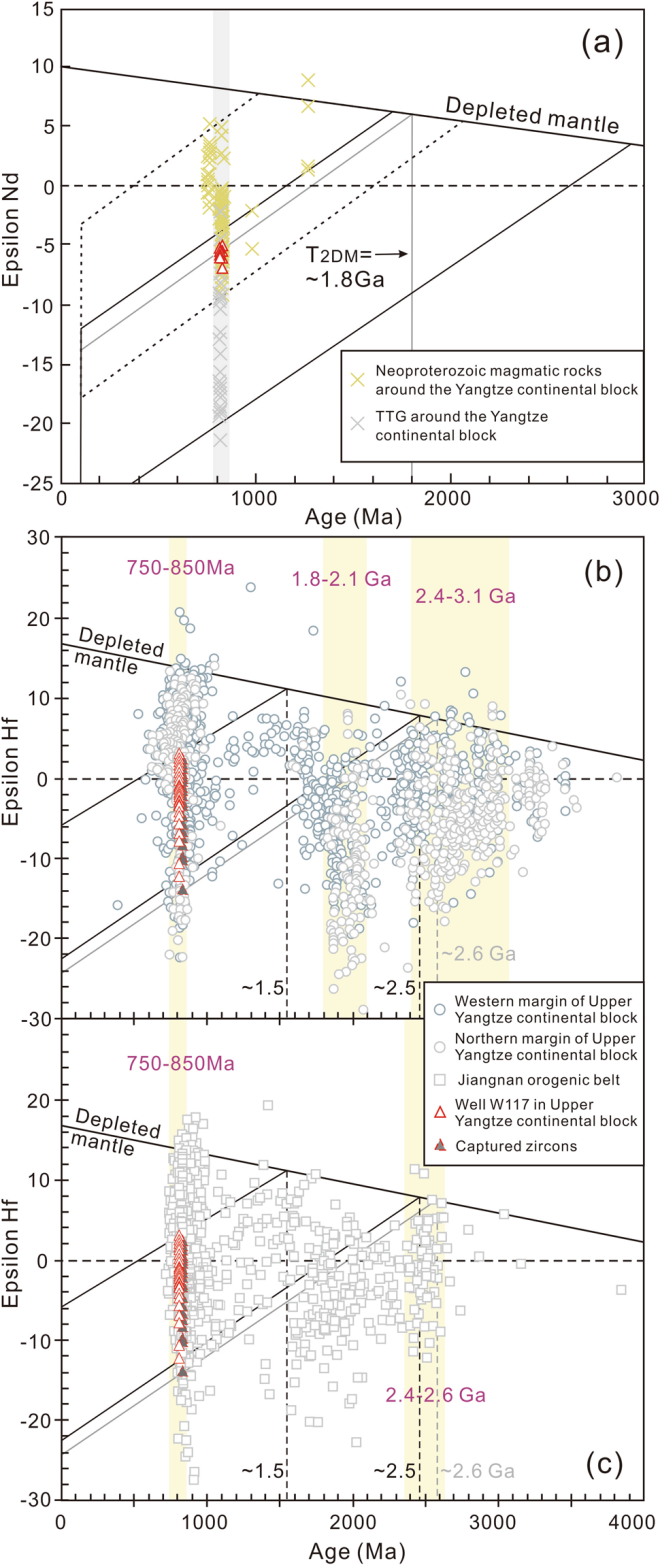
(**a**) Whole rock *ε*_Nd_(*t*) values versus U–Pb age diagram and (**b**) Zircon *ε*_Hf_(*t*) values versus U–Pb age diagram of the quartz monzonites from Well W117. The compared whole rock Nd isotopic data are from Li *et al*.^2^, Ge *et al*.^51^, Zhang *et al*.^52^, Zhou *et al*.^76^, Zhou *et al*.^77^ and Wang *et al*.^78^, and zircon Lu–Hf isotopic data from the northern and western Yangtze continental block and the Jiangnan Orogenic Belt are from Wang *et al*.^44,79,80^.

Measured whole rock Sr–Nd and zircon Hf isotopic data for the samples are reported in Table 3S and 4S. The initial ε_Nd_(t) and ε_Hf_(t) values were determined based on the calculated formation ages (ca. 814 Ma). The calculated results suggest that both types of quartz monzonites from Well W117 exhibit similar whole rock Sr–Nd isotopic compositions. They have very high initial ^87^Sr/^86^Sr ratios (0.9441–1.3309, Table S3), and negative ε_Nd_(t) values (−5.2 to −7.0; Fig. 5a), with two-stage (T_2DM_) Nd model ages from 1.76 to 1.87 Ga. Extremely high initial ^87^Sr/^86^Sr ratios of the samples are likely attributed to weak alteration that resulted in modification of the contents of Rb and Sr mobile elements^50^. These quartz monzonites plot below the CHUR line in the Sr–Nd isotopic diagram (Fig. 5a), overlapping the TTG complex and other magmatic rocks cropping out in the Upper Yangtze continental block^2,51,52^.

All of the analyzed zircons have low ^176^Lu/^177^Hf ratios (0.000890–0.003211), with an average value of 0.001562, indicating limited radiogenic Hf production over their lifetime. Thirty-four zircon grains from the Well W117 coarse-grained quartz monzonites (samples 117-4 and 117-5) have initial ^176^Hf/^177^Hf ratios between 0.281943 and 0.282368, with variable ε_Hf_(t) values (−12.3 to 2.3) and two-stage Hf model ages, ranging from 1.55 Ga to 2.48 Ga (Fig. 5b,c; Table S4). Twenty-four captured zircon grains from fine-grained quartz monzonites (117-3) yielded initial ^176^Hf/^177^Hf ratios ranging from 0.281894 to 0.282341, corresponding to a wide range of ε_Hf_(t) values (−13.9 to 2.0) and two-stage Hf model ages (1.59–2.59 Ga) (Fig. 5b,c; Table S4).

## Discussion and Conclusions

### Genetic type of the Neoproterozoic quartz monzonites

The Well W117 quartz monzonites are metaluminous to weakly peraluminous with A/CNK ratios ranging from 0.93 to 1.12, and have relatively high TFe_2_O_3_/(TFe_2_O_3_ + MgO) (0.66–0.92), 10000 Ga/Al (2.47–5.37) and moderate to high Ce + Nb + Zr + Y contents, analogous to those of typical A-type granites^48^. This seems to be also supported by high Rb and low Sr concentrations. However, zircon saturation thermometer suggests that these samples formed at low temperatures from 831 °C to 650 °C (mostly < 800 °C) (Table 2S), following the method of Watson and Harrison^53^, making them remarkable different from ferroan magma (normally >800 °C)^54^. Instead, the Well W117 quartz monzonites possess high SiO_2_ (72.26–77.93%), total alkali abundance and TFe_2_O_3_/MgO (2.93–12.2) ratio, and low CaO content (0.19–1.16%) and Zr/Hf ratio (18.2–31.1). No amphibole has been observed in these quartz monzonite samples under the optical microscope. These features, combined with low total REE content and “tetrad effect” phenomenon, indicate that the Well W117 quartz monzonites experienced a high degree of fractional crystallization^55,56^, corresponding to highly fractionated granites. In addition, the Zr contents of the quartz monzonites show a decrease with increasing 10000 Ga/Al ratios (Fig. 4c), providing further evidence in support of strong fractionation^57^. It is noteworthy that the quartz monzonites contain relatively low P_2_O_5_ content (0.04–0.11%), which display a negative correlation with SiO_2_ content (Fig. 4d). This trend is typical of I-type granites rather than S-type ones, because apatite reaches saturation in metaluminous to weakly peraluminous magmas, but is highly soluble in peraluminous melts^58^. The above information allows us to classify the quartz monzonites from Well W117 as highly fractionated I-type granites.

### Source and formation of the Neoproterozoic quartz monzonites

**Figure 6:**
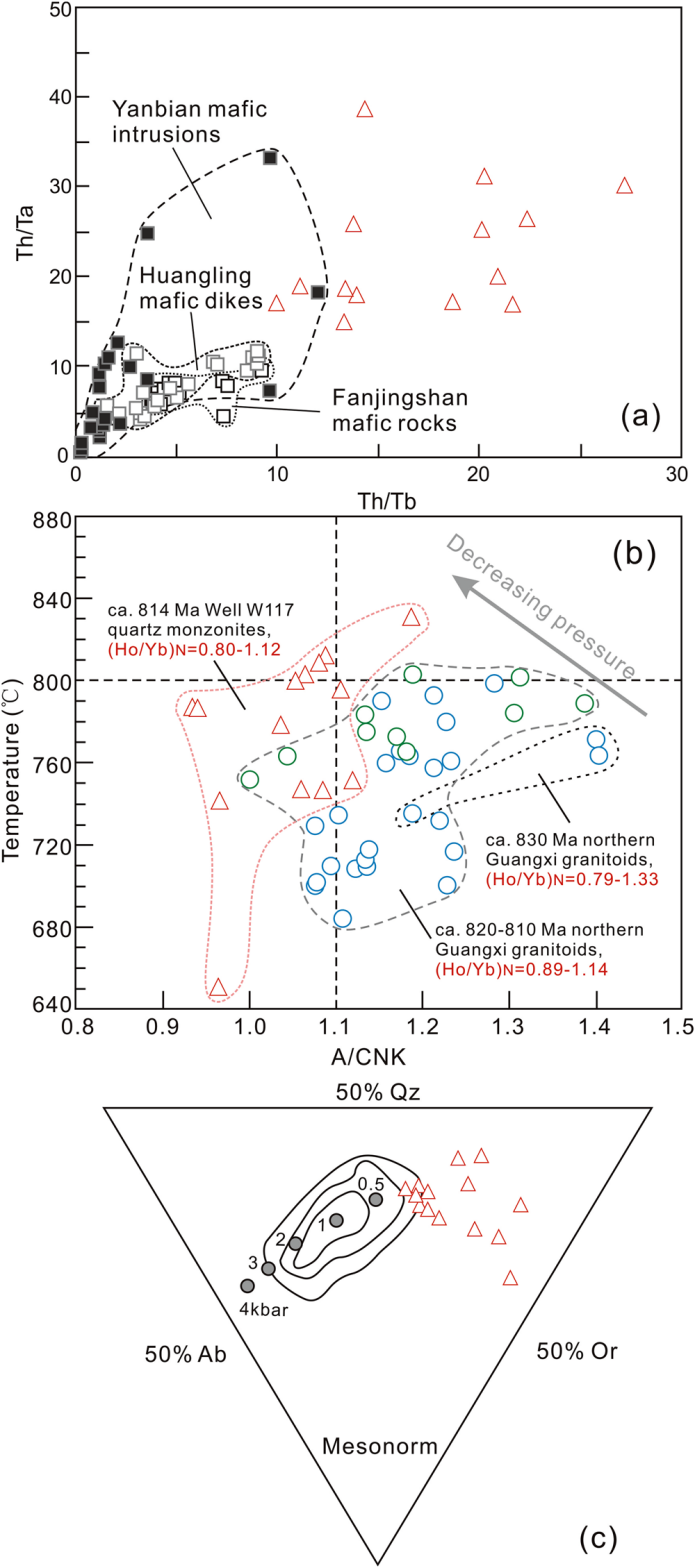
(**a**) Th/Ta versus Th/Yb diagram showing the correlation between Huangling mafic dikes^25^, Yanbian maifc intrusions^5^ and Fanjingshan mafic rocks^59^, and Well W117 quartz monzonites. (**b**) Diagram of zircon saturation temperatures versus A/CNK ratios. The temperatures were estimated using zircon saturation thermometry^53^. The compared data for the granitoids are from Li *et al*.^2^ and Yao *et al*.^17^. (**c**) Barth mesonormative Qz–Ab–Or ternary diagram for the granitic rock. Contour diagram of mesonormative Qz, Ab and Or molecules of the plutonic rocks and its relationship to the pressure are after Parslow^81^.

The Neoproterozoic quartz monzonites from Well W117 exhibit subparallel incompatible trace element patterns (Fig. 4e,f) and a restricted ε_Nd_(t) values (−7.0 to −5.2; Fig. 5a). These features, together with the lack of correlation between the ε_Nd_(t) values and constant SiO_2_ abundance, imply that the geochemical variability and isotopic compositions of these rocks may be mainly controlled by the source processes rather than possible crustal contamination. The ε_Nd_(t) values of the quartz monzonites are distinctly lower than those of the coeval Yanbian mafic intrusions (ε_Nd_(t) = 1.5 to 6.0^5^) and Fanjingshan mafic rocks (ε_Nd_(t) = −4.16 to −0.41^59^) that represent the isotopic compositions of mid-Neoproterozoic lithospheric mantle source^5,59^, indicating that these samples may not have been produced simply by lithospheric mantle-derived magmas. Alternatively, considering the facts that their epsilon Nd values overlap with those of Huangling mafic dikes that derived from contaminated lithospheric mantle by crust material (−10.9 to −4.2^25^), and that the majority of the quartz monzonite samples have low Mg# values (<40) and high Rb/Sr ratios (up to 32) (Table 2S), we suggest that continental crust may have contributed to the source of the Well W117 quartz monzonites. Zircon Lu–Hf isotopes can be used to further clarify the magma source and to identify the involvement of evolved crust components^60^. The Well W117 quartz monzonites display a broad range of zircon ε_Hf_(t) values, from −12.3 to 2.3 (Table 4S), reflecting that both of juvenile material and ancient crust are incorporated into their magma sources. The ~814 Ma and captured (~830 Ma) zircons from the quartz monzonite samples show Hf crustal model ages of ca. 1.5–2.5 Ga with an age peak of 1.7–2.0 Ga (Fig. 5b,c; Table 4S), which is consistent with the whole rock Nd crustal model ages (~1.8 Ga, Fig. 5a), implying that the reworked juvenile materials are represented primarily by Paleoproterozoic newly-formed crust. The existence of zircons with the lowest ε_Hf_(t) values (−14 to −12) mirrors the involvement of Neoarchean (~2.5–2.6 Ga) or more ancient crust in the formation of the Well W117 quartz monzonites. Th/Ta and Th/Tb ratios can provide important constraints on the origin and evolution of mafic magmas^61^. The quartz monzonites do not display any linear correlation with those contemporaneous mafic magmas around the Yangtze area (Fig. 6a), indicating insignificant input of mantle-derived melts into the Well W117 quartz monzonite magmas. Thus, we propose that the Well W117 quartz monzonites were more probably generated by mixing of newly-formed crust-derived melts and ancient crustal materials.

As mentioned above, most of the Well W117 quartz monzonites were derived from magmas with low temperatures. However, it should be noted that a few samples record a relatively high temperature condition (up to 831 °C, Fig. 6b). This result, coupled with the presence of extensive mafic magmatism at ca. 820–810 Ma in the Yangtze area, suggests that mantle material exerted an important effect on the formation of the borehole granites. These mantle-derived magmas could provide the heat that triggered the partial melting of a crustal magma source. Quartz crystals in the Well W117 quartz monzonites are mostly xenomorphic, indicating that they crystallized later than other mineral components, and suggesting that the granites were emplaced in a low pressure context. The samples mainly plot in the low pressure field (approximately of 2–4 kb) of the Barth mesonormative Qz–Ab–Or diagram (Fig. 6c), with an average value of 3 kb, corresponding to an emplacement depth of 9–10 km below the surface. Therefore, we propose that the parental melts of the Well W117 quartz monzonites were produced as a result of partial melting of co-existing ancient and juvenile crust triggered by hot mantle-derived magmas. Subsequently, the melts experienced highly fractionation with decreasing temperature until their emplacement.

### Cratonic evolution and geodynamic implications

New seismic profiles revealed some Neoproterozoic extensional structures beneath the Sichuan Basin^35,62^, implying that the Upper Yangtze continental block underwent rifting event at that time, although such a block usually resists deformation and fragmentation due to its cratonic rigidity. However, the formation time and mechanics of this period of rifting event remain unclear so far. The drilled quartz monzonites and overlying sedimentary sequences by Well W117 in the rift basin provide new insights to the evolution processes of the Yangtze Craton. The dating result for the crystallized and captured zircons from the borehole quartz monzonites constrains the formation time of the rift to be no later than ca. 814 Ma, with a possible initial time of ca. 830 Ma (Fig. 3). The ca. 814 Ma rift-related quartz monzonites were originally emplaced into the shallow crust at a depth of 9–10 km. In view of this tectonic history and the present thickness of the crust beneath the Sichuan Basin (42 km^63^) with ~3.5–4 km sedimentary cover, as well as the lack of evidence for significant crustal growth or large-scale imbricate thrust systems in the interior of the Upper Yangtze Craton after the Proterozoic, we infer that the Upper Yangtze Craton likely had a crustal thickness of ~38 km during the magma emplacement. Afterward, it experienced cooling and subsidence during the middle-to-late Neoproterozoic, as evidenced by the tectono-sedimentary records that the quartz monzonites are unconformably overlain by Sinian (late Neoproterozoic) sag basin sediments. The Neoproterozoic rift sequences in the craton exhibit approximately horizontal or concave upward-type seismic reflectors^62^, ruling out the later structural inversion. Taking into account the continuous development of mid-Neoproterozoic rift magmas and/or successions around the Upper Yangtze Craton, it is suggested that the extension was more likely responsible for the cooling, uplift and 9–10 km crustal denudation of the craton which resulted in the exhumation of quartz monzonites. Such a tectonic scenario marks a mid-Neoproterozoic rifting process of the Upper Yangtze Craton from rift to sag since ca 814 Ma.

**Figure 7:**
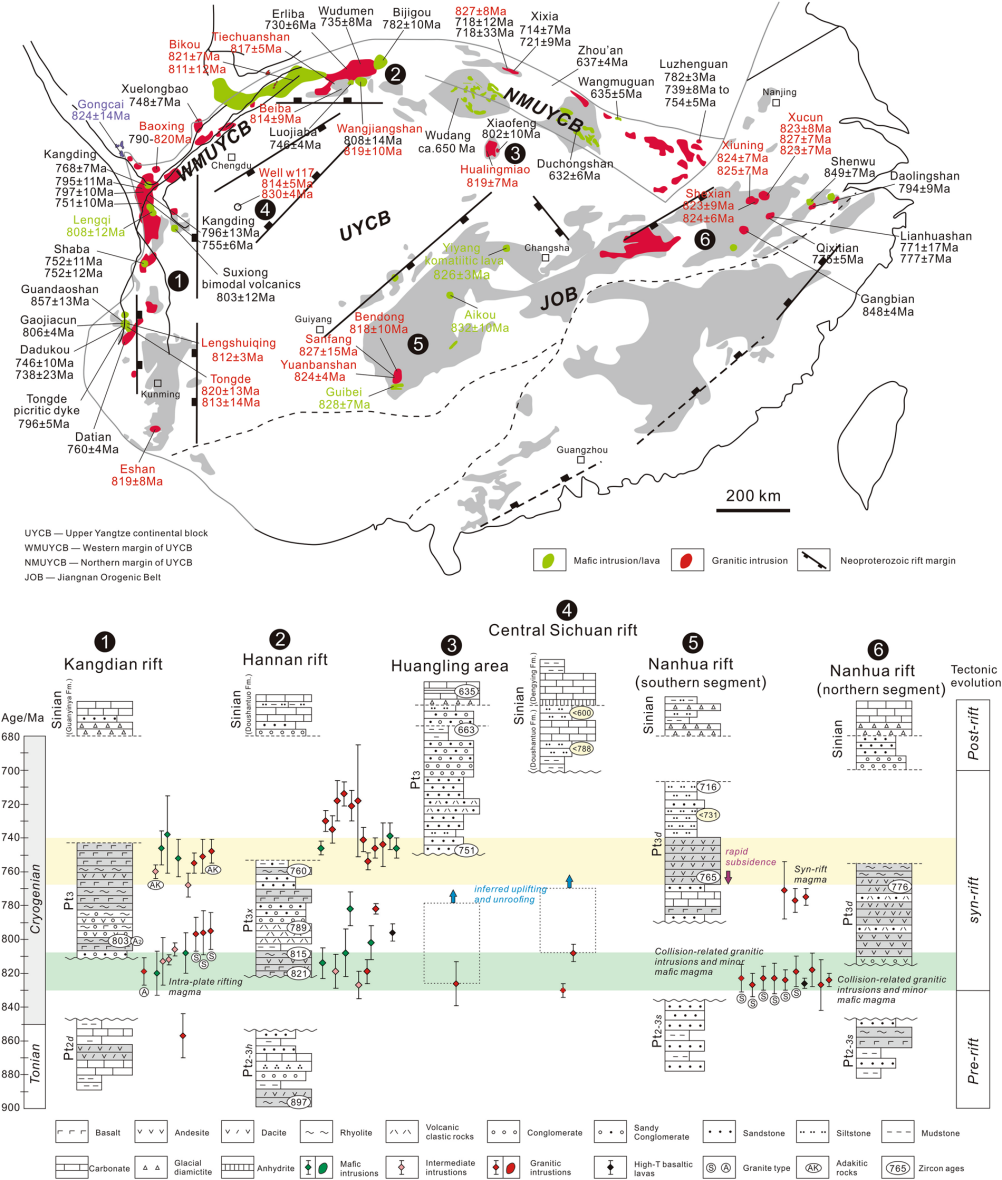
Generalized space–time diagram showing the distribution of middle Neoproterozoic stratigraphic successions and magmatic bodies in the Upper Yangtze continental block^21,24,32,44,80^. Pt_2_ *d*–Meso-proterozoic Dengxiangying Group; Pt_2-3_ *h*–Meso- to Neo-proterozoic Huodiya Group; Pt_2-3_ *s*–Meso- to Neo-proterozoic Sibao Group; Pt_3_ *x*–Neo-proterozoic Xixiang Group; Pt_3_ *d*–Neo-proterozoic Danzhou Group. This figure is generated by Di Li, using CorelDRAW X6 created by the CorelDRAW Team under an open license (http://www.coreldraw.com/cn/product/graphic-design-software/).

The Neoproterozoic rifting and associated magmatism and evolution processes of the Upper Yangtze Craton provide some constraints on the tectonics of the whole Yangtze area. The Well W117 quartz monzonites show high SiO_2_ and total alkali contents and variable A/CNK ratios, resembling those coeval collision-related granitoids (e.g., Sanfang, Bendong and Yuanbashan granites) in the western part of the Jiangnan Orogenic Belt^2,17^. Their formation had been correlated to reworking of the juvenile and ancient crust^4^. It is noted that the Well W117 quartz monzonites display much lower ε_Nd_(t) values compared to the granitoids in the western part of the Jiangnan Orogenic Belt (−5.0 to −5.8^64,65^), indicating relatively less proportions of juvenile crust component in protoliths in the interior of the craton. The chondrite-normalized (Ho/Yb)_N_ ratio in the granitic magma, mainly affected by residue of garnet during the partial melting process, can be used to limit the melting pressure^66^. The results suggest a ca. 830–810 Ma melting condition of increase temperature and decrease pressure with time plus the zircon saturation temperatures (Fig. 6b), implying that the rifting resulted in crust extension during that period. Given that the Well W117 quartz monzonites display typical features of post-collisional granites elsewhere in the world with high K_2_O, Ba and Sr, but relatively low Nb and Ta abundances^67,68^ (Fig. 4), and that the NE-trending Neoproterozoic rift structures in the basin basement are approximately parallel to the Jiangnan Orogenic Belt, we propose that the Well W117 quartz monzonites and other ca. 820–810 Ma magmas most likely occurred in a post-collisional setting, following the collision between the Upper Yangtze and Cathaysia continental blocks^26,69^ (Fig. 7). It should be noted that the occurrence of numerous mafic dikes and high-T basalts around the periphery of the Upper Yangtze Craton has been interpreted to suggest that a mantle plume played an important role in the mid-Neoproterozoic tectonic evolution of the South China^70–72^. Nevertheless, no radiate pattern as the center of the Upper Yangtze Craton is apparent in the distribution of the rift zones (Fig. 7), which does not seem to support the mantle plume model for explaining the formation of the ca 820–810 Ma magmas. Even if it did play a role, the position of its plume head may have been far from the Upper Yangtze Craton at that time. In contrast, the ~750 Ma continental rifting and related magmatism probably resulted from a mantle plume (Fig. 7), as evidenced by the presence of high temperature picrites and Guibei spilites near the Upper Yangtze Craton^71^. Importantly, this interpretation is supported by the unroofing of the craton after the emplacement of the Well W117 quartz monzonite, as suggested by their exhumation.

**Figure 8:**
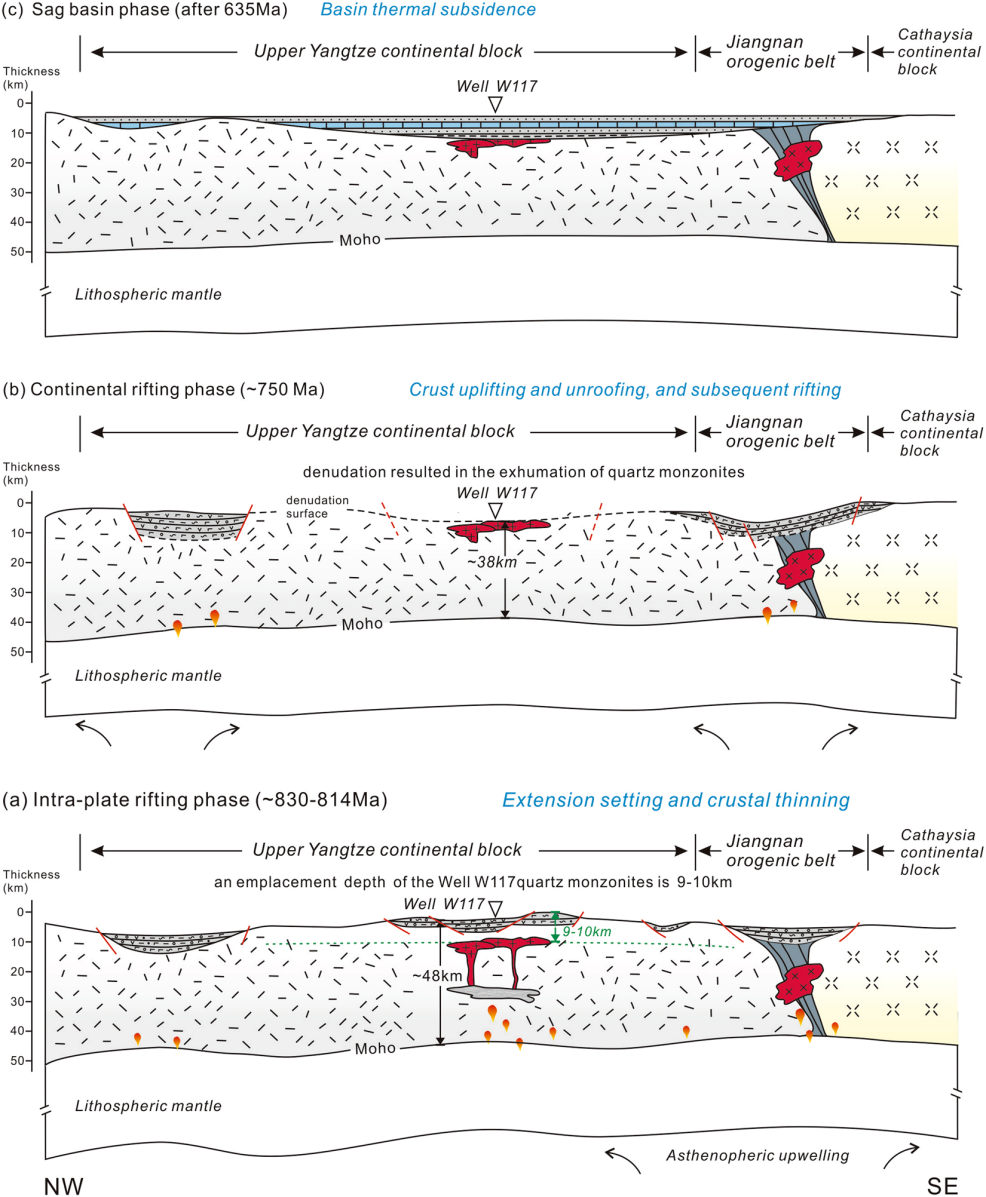
Schematic diagrams showing the middle Neoproterozoic tectonic framework of the Upper Yangtze continental block and the evolution of the Sichuan Basin. See text for details. This figure is generated by Di Li, using CorelDRAW X6 created by the CorelDRAW Team under an open license (http://www.coreldraw.com/cn/product/graphic-design-software/).

Given the above discussion, the rifting processes and geodynamic evolution of the Upper Yangtze continental block can be divided into three phases as follows: 1) during 830–814 Ma, reworking of the Upper Yangtze continental block was mainly controlled by post-collisional extension, which promoted the development of voluminous rift structures and related magmas, including the Well W117 quartz monzonites in the Sichuan basin basement (Fig. 8a); 2) the block underwent crustal uplift and exhumation in an active rift setting resulting from a ~750 Ma mantle plume, with an eroded thickness of the upper crust up to 9–10 km (Fig. 8b); 3) the Upper Yangtze continental block began to subside and to be buried by sag basin sediments since 635 Ma (Fig. 8c).

Our work suggests that the basement underlying the Sichuan basin underwent a cooling process following the ~814 Ma post-collisional extension, leading to 9–10 km of shallow crustal denudation, and shed light on two phases of rifting in the Upper Yangtze continental block during the middle Neoproterozoic (830–635 Ma), possibly associated with the breakup of the Rodinia supercontinent.

## Methods

### Zircon LA-ICP-MS U–Pb dating

Zircon grains in the Well W117 quartz monzonites (117-3 and 117-4) were separated for LA-ICP-MS analysis using conventional heavy liquid and magnetic techniques. Representative grains were hand picked using a binocular microscope, mounted in an epoxy resin disk, and then polished and coated with a gold film. The grains were imaged under transmitted and reflected light micrographs as well as cathodoluminescence (CL) to reveal their internal structures, and the mount was vacuum-coated with high purity gold. Measurements of U, Th and Pb were conducted using a multi-collector-inductively coupled plasma-mass spectrometer (MC-ICP-MS) at the Institute of Mineral Resources, Chinese Academy of Geological Sciences (CAGS), Beijing, China. The zircons GJ-1, M127, and Plešovice were used as standards during the analyses. The detailed analytical procedures are described by Hou *et al*.^82^. The U–Pb ages were calculated and plotted using the software Isoplot/Ex ver. 3.0^83^.

### Major and trace element analyses

Major and trace elements of the quartz monzonite samples from well W117 were carried out in the Analytical Laboratory of the Beijing Research Institute of Uranium Geology. Major elements were analyzed by a Philips PW2404 X-ray fluorescence spectrometer (XRF). Trace element data were obtained using a Finnigan MAT high resolution inductively coupled plasma mass spectrometer (HR-ICPMS). The precision and accuracy of the ICP-MS and X-ray fluorescence data were reported by Cullen *et al*.^84^ and Wu *et al*.^85^, respectively.

### Whole rock Sr–Nd analyses

Sr-Nd isotopic data of quartz monzonites were generated at the Institute of Geology and Geophysics, Chinese Academy of Sciences. The Sr isotope compositions were measured by isotope dilution on a Finnigan MAT-262 mass spectrometer. The Nd isotope compositions were acquired with a Nu Plasma HR multi-collector inductively coupled plasma mass spectrometry (MC-ICP-MS). Procedural details are described by Zhang *et al*.^86^.

### *In-situ* Lu–Hf isotopic analyses

*In-situ* zircon Hf isotopic analyses of the Well W117 quartz monzonites (117-3, 117-4 and 117-5) were conducted on the same spots where U–Pb analyses were made. Hf isotopic compositions were determined by a Neptune MC-ICP-MS equipped with Geolas Plus 193 nm ArF excimer laser at the Institute of Geology and Geophysics, Chinese Academy of Sciences. A laser spot size of 44 μm and a laser repetition of 8 Hz with energy density of 15 J/cm^2^ were used during the analyses. The signal collection model was one block with 200 cycles, with an integration time of 0.131 s for 1 cycle and a total time of 26 s during each analysis. Zircon 91500 was used as external standard for Hf isotopic analyses and was analyzed twice every 5 analyses. Replicate analyses of 91500 yielded a mean ^176^Hf/^177^Hf ratio of 0.282300 ± 24 (2σ, n = 82), which is concordant with the^176^Hf/^177^Hf ratios, measured by Goolaerts *et al*.^87^.

## Electronic supplementary material

Dataset 1
