# Supplementary material for: Neoproterozoic rifting in the Upper Yangtze Continental Block: Constraints from granites in the Well W117 borehole, South China
Source: Sci Rep. 2017 Oct 2;7:12542. doi: 10.1038/s41598-017-12764-y (PMC5624896; doi:10.1038/s41598-017-12764-y)
Supplement: Supplementary file 1 — Dataset 1 [file 41598_2017_12764_MOESM1_ESM.doc]

**Online supplementary information**

**Neoproterozoic rifting in the Upper Yangtze Continental Block: Constraints from granites in the Well W117 borehole, South China**

**Deng-Fa He*, Di Li*, Chuan-Xin Li, Ying-Qiang Li, Qing-Hua Mei**

***Corresponding author,**

**Email:** [**hedengfa282@263.net**](mailto:hedengfa282@263.net) **(D.F.H.);** [**xdlidi@163.com**](mailto:xdlidi@163.com) **(D.L.)**

**Includes the following materials:**

**1. Figure S1 Cathodoluminescence (CL) images of the analyzed zircon grains from the Well W117 granites in the central Sichuan Basin**

**2. Geochronological, geochemical and isotopic data**

**Table S1** **Results of LA-ICP-MS U–Pb dating of zircons from the Well W117 quartz monzonites in the central Sichuan Basin**

**Table S2 Results of major and trace element analysis of the Well W117 quartz monzonites in the central Sichuan Basin.**

**Note:** LOI=Loss on ignition; Mg#=Mg^2+^/(Mg^2+^+Fe^2+^)×100; Eu/Eu*=Eu_N_/(Sm_N_×Gd_N_)^1/2^; A/NK=molar Al_2_O_3_/(Na_2_O+K_2_O); A/CNK=molar Al_2_O_3_/(Na_2_O+K_2_O+CaO); N=chondrite-normalized data

**Table S3 Whole–rock Sr–Nd isotopic compositions of the Well W117 quartz monzonites in the central Sichuan Basin.**

**Note:***T*_2DM_=(1/λ)ln(1+((^143^Nd/^144^Nd)_S_−(^143^Nd/^144^Nd)_DM_−((^147^Sm/^144^Nd)_S_−(^147^Sm/^144^Nd)_C_(e^λt^−1))/((^147^Sm/^144^Nd)_C_−(^147^Sm/^144^Nd)_DM_)).

**Table S4 Results of Lu–Hf isotopic analysis of zircons from the Well W117 quartz monzonites in the central Sichuan Basin**

**Note:** ^176^Lu decay constant is 1.865×10^−11^ yr^−1^;

Chondritic values: ^176^Lu/^177^Hf =0.0332±0. 0002, ^176^Hf/^177^Hf =0. 282772±0.000029;

Depleted mantle values: (^176^Lu/^177^Hf)_DM_=0.0384, (^176^Hf/^177^Hf)_DM_=0.28325;

*T*_DM1_=1/λ×ln(1+((^176^Hf/^177^Hf)_S_−(^176^Hf/^177^Hf)_DM_)/((^176^Lu/^177^Hf)_S_−(^176^Lu/^177^Hf)_DM_));

*T*_DM2_=1/λ×ln(1+((^176^Hf/^177^Hf)_S,t_−(^176^Hf/^177^Hf)_DM,t_)/((^176^Lu/^177^Hf)_S_−(^176^Lu/^177^Hf)_DM_))+t

**Neoproterozoic rifting in the Upper Yangtze Continental Block: Constraints from granites in the Well W117 borehole, South China**

Deng-Fa He*, Di Li*, Chuan-Xin Li, Ying-Qiang Li, Qing-Hua Mei

**
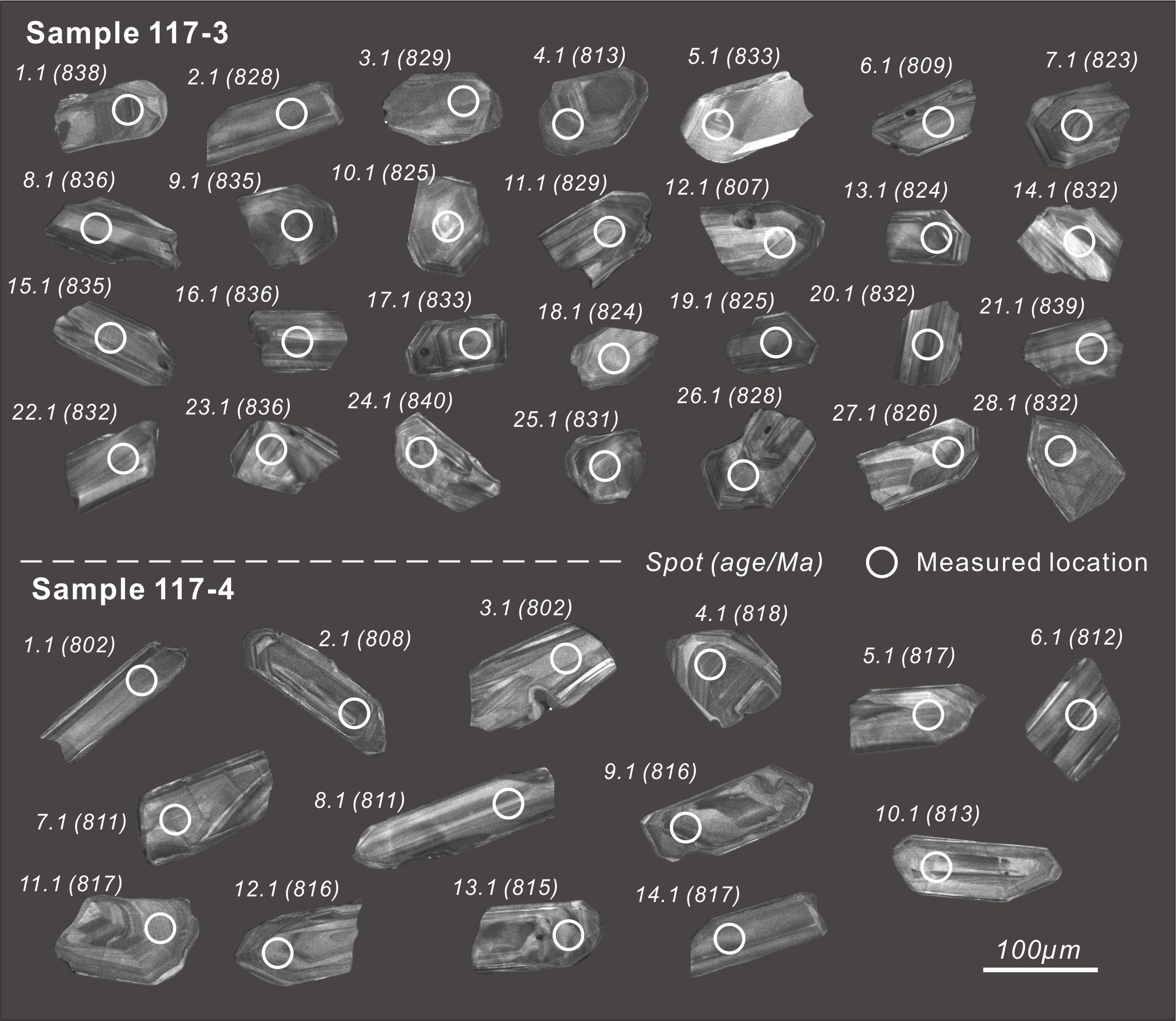
**

**Figure S1 Cathodoluminescence (CL) images of the analyzed zircon grains from the Well W117 quartz monzonites**

**Neoproterozoic rifting in the Upper Yangtze Continental Block: Constraints from granites in the Well W117 borehole, South China**

Deng-Fa He*, Di Li*, Chuan-Xin Li, Ying-Qiang Li, Qing-Hua Mei

**Table S1** **Results of LA-ICP-MS U–Pb dating of zircons from the Well W117 quartz monzonites in the central Sichuan Basin**

| Spot | Th/U | Isolopic contents（ppm） | | Isolopic ratios | | | | | | Apparent age（Ma） | | | | | |
| --- | --- | --- | --- | --- | --- | --- | --- | --- | --- | --- | --- | --- | --- | --- | --- |
|  |  | ^232^Th | ^238^U | ^207^Pb/^206^Pb | | ^207^Pb/^235^U | | ^206^Pb/^238^U | | ^207^Pb/^206^Pb | | ^207^Pb/^235^U | | ^206^Pb/^238^U | |
|  |  |  |  |  | 1s |  | 1s |  | 1s | Age | 1s | Age | 1s | Age | 1s |
| Sample 117-3 | |  |  |  |  |  |  |  |  |  |  |  |  |  |  |
| 1.1 | 1.26 | 365 | 290 | 0.0676 | 0.0016 | 1.2952 | 0.0312 | 0.1388 | 0.0014 | 857 | 47 | 844 | 14 | 838 | 8 |
| 2.1 | 0.80 | 143 | 177 | 0.0709 | 0.0028 | 1.3420 | 0.0544 | 0.1370 | 0.0018 | 955 | 81 | 864 | 24 | 828 | 10 |
| 3.1 | 0.37 | 149 | 408 | 0.0703 | 0.0016 | 1.3268 | 0.0317 | 0.1372 | 0.0022 | 937 | 42 | 857 | 14 | 829 | 12 |
| 4.1 | 0.89 | 201 | 226 | 0.0671 | 0.0028 | 1.2389 | 0.0541 | 0.1344 | 0.0030 | 843 | 86 | 818 | 25 | 813 | 17 |
| 5.1 | 0.71 | 198 | 279 | 0.0672 | 0.0045 | 1.2785 | 0.0883 | 0.1380 | 0.0068 | 843 | 139 | 836 | 39 | 833 | 39 |
| 6.1 | 0.76 | 251 | 331 | 0.0671 | 0.0016 | 1.2310 | 0.0321 | 0.1338 | 0.0020 | 843 | 55 | 815 | 15 | 809 | 11 |
| 7.1 | 1.23 | 281 | 229 | 0.0686 | 0.0029 | 1.2832 | 0.0550 | 0.1362 | 0.0029 | 887 | 88 | 838 | 24 | 823 | 17 |
| 8.1 | 0.44 | 221 | 503 | 0.0677 | 0.0015 | 1.2909 | 0.0299 | 0.1385 | 0.0019 | 859 | 46 | 842 | 13 | 836 | 11 |
| 9.1 | 0.61 | 216 | 357 | 0.0697 | 0.0040 | 1.3203 | 0.0669 | 0.1383 | 0.0041 | 920 | 117 | 855 | 29 | 835 | 23 |
| 10.1 | 2.10 | 570 | 272 | 0.0693 | 0.0025 | 1.3057 | 0.0486 | 0.1366 | 0.0025 | 909 | 79 | 848 | 21 | 825 | 14 |
| 11.1 | 0.59 | 166 | 282 | 0.0681 | 0.0047 | 1.2727 | 0.0716 | 0.1372 | 0.0061 | 872 | 144 | 834 | 32 | 829 | 35 |
| 12.1 | 0.99 | 236 | 239 | 0.0657 | 0.0032 | 1.2196 | 0.0723 | 0.1334 | 0.0038 | 796 | 97 | 810 | 33 | 807 | 21 |
| 13.1 | 1.49 | 344 | 231 | 0.0678 | 0.0027 | 1.2646 | 0.0467 | 0.1363 | 0.0024 | 865 | 77 | 830 | 21 | 824 | 14 |
| 14.1 | 0.62 | 141 | 226 | 0.0668 | 0.0024 | 1.2723 | 0.0504 | 0.1377 | 0.0025 | 831 | 73 | 833 | 23 | 832 | 14 |
| 15.1 | 0.74 | 245 | 329 | 0.0680 | 0.0028 | 1.3016 | 0.0595 | 0.1383 | 0.0029 | 870 | 85 | 846 | 26 | 835 | 16 |
| 16.1 | 1.05 | 226 | 215 | 0.0689 | 0.0036 | 1.2998 | 0.0563 | 0.1384 | 0.0035 | 894 | 107 | 846 | 25 | 836 | 20 |
| 17.1 | 0.54 | 299 | 558 | 0.0669 | 0.0014 | 1.2752 | 0.0275 | 0.1379 | 0.0015 | 835 | 44 | 835 | 12 | 833 | 9 |
| 18.1 | 1.59 | 269 | 169 | 0.0672 | 0.0027 | 1.2603 | 0.0498 | 0.1364 | 0.0025 | 856 | 84 | 828 | 22 | 824 | 14 |
| 19.1 | 1.24 | 255 | 206 | 0.0668 | 0.0020 | 1.2550 | 0.0366 | 0.1366 | 0.0019 | 831 | 61 | 826 | 16 | 825 | 11 |
| 20.1 | 2.11 | 266 | 126 | 0.0722 | 0.0027 | 1.3654 | 0.0493 | 0.1378 | 0.0018 | 991 | 75 | 874 | 21 | 832 | 10 |
| 21.1 | 1.12 | 224 | 200 | 0.0682 | 0.0077 | 1.3142 | 0.1589 | 0.1390 | 0.0039 | 874 | 236 | 852 | 70 | 839 | 22 |
| 22.1 | 1.31 | 251 | 192 | 0.0681 | 0.0041 | 1.2907 | 0.0754 | 0.1377 | 0.0027 | 872 | 124 | 842 | 33 | 832 | 15 |
| 23.1 | 0.44 | 166 | 373 | 0.0683 | 0.0014 | 1.3078 | 0.0286 | 0.1384 | 0.0014 | 880 | 43 | 849 | 13 | 836 | 8 |
| 24.1 | 1.10 | 167 | 152 | 0.0695 | 0.0032 | 1.3331 | 0.0605 | 0.1391 | 0.0020 | 922 | 93 | 860 | 26 | 840 | 11 |
| 25.1 | 0.87 | 226 | 259 | 0.0678 | 0.0020 | 1.2827 | 0.0366 | 0.1376 | 0.0017 | 861 | 61 | 838 | 16 | 831 | 10 |
| 26.1 | 1.00 | 349 | 347 | 0.0670 | 0.0016 | 1.2680 | 0.0315 | 0.1370 | 0.0014 | 837 | 50 | 831 | 14 | 828 | 8 |
| 27.1 | 0.59 | 162 | 277 | 0.0667 | 0.0017 | 1.2571 | 0.0324 | 0.1367 | 0.0014 | 828 | 54 | 827 | 15 | 826 | 8 |
| 28.1 | 0.83 | 234 | 283 | 0.0666 | 0.0020 | 1.2696 | 0.0392 | 0.1377 | 0.0018 | 828 | 61 | 832 | 18 | 832 | 10 |
| Sample 117-4 | |  |  |  |  |  |  |  |  |  |  |  |  |  |  |
| 1.1 | 1.05 | 315 | 300 | 0.0693 | 0.0058 | 1.2551 | 0.0947 | 0.1326 | 0.0035 | 907 | 173 | 826 | 43 | 802 | 20 |
| 2.1 | 0.89 | 288 | 323 | 0.0715 | 0.0055 | 1.3202 | 0.1043 | 0.1335 | 0.0031 | 972 | 156 | 855 | 46 | 808 | 18 |
| 3.1 | 1.16 | 330 | 285 | 0.0675 | 0.0033 | 1.2263 | 0.0559 | 0.1325 | 0.0021 | 854 | 101 | 813 | 26 | 802 | 12 |
| 4.1 | 1.14 | 308 | 270 | 0.0670 | 0.0023 | 1.2571 | 0.0461 | 0.1354 | 0.0016 | 839 | 71 | 827 | 21 | 818 | 9 |
| 5.1 | 1.73 | 440 | 255 | 0.0670 | 0.0026 | 1.2464 | 0.0464 | 0.1352 | 0.0018 | 839 | 75 | 822 | 21 | 817 | 10 |
| 6.1 | 2.12 | 899 | 424 | 0.0669 | 0.0024 | 1.2381 | 0.0433 | 0.1342 | 0.0015 | 835 | 71 | 818 | 20 | 812 | 9 |
| 7.1 | 1.73 | 559 | 323 | 0.0664 | 0.0019 | 1.2261 | 0.0367 | 0.1341 | 0.0019 | 820 | 56 | 813 | 17 | 811 | 11 |
| 8.1 | 1.76 | 341 | 194 | 0.0703 | 0.0024 | 1.2883 | 0.0427 | 0.1341 | 0.0018 | 939 | 70 | 841 | 19 | 811 | 10 |
| 9.1 | 1.36 | 356 | 262 | 0.0686 | 0.0020 | 1.2744 | 0.0370 | 0.1350 | 0.0014 | 887 | 56 | 834 | 17 | 816 | 8 |
| 10.1 | 0.98 | 161 | 165 | 0.0729 | 0.0042 | 1.3360 | 0.0701 | 0.1344 | 0.0024 | 1013 | 112 | 861 | 30 | 813 | 13 |
| 11.1 | 1.49 | 444 | 297 | 0.0680 | 0.0021 | 1.2571 | 0.0395 | 0.1350 | 0.0019 | 878 | -134 | 827 | 18 | 817 | 11 |
| 12.1 | 2.16 | 475 | 220 | 0.0720 | 0.0020 | 1.3390 | 0.0384 | 0.1349 | 0.0015 | 987 | 57 | 863 | 17 | 816 | 8 |
| 13.1 | 0.75 | 314 | 417 | 0.0676 | 0.0017 | 1.2539 | 0.0296 | 0.1347 | 0.0013 | 857 | 52 | 825 | 13 | 815 | 7 |
| 14.1 | 0.65 | 339 | 522 | 0.0717 | 0.0034 | 1.3311 | 0.0521 | 0.1351 | 0.0041 | 989 | 96 | 859 | 23 | 817 | 23 |

**Neoproterozoic rifting in the Upper Yangtze Continental Block: Constraints from granites in the Well W117 borehole, South China**

Deng-Fa He*, Di Li*, Chuan-Xin Li, Ying-Qiang Li, Qing-Hua Mei

**Table S2 Results of major and trace element analysis of the Well W117 quartz monzonites in the central Sichuan Basin.**

| Sample | 117-1 | 117-2 | 117-3 | 117-39 | 117-4 | 117-5 | 117-20 | 117-40 | W117_1* | W117_2* | W117_3* | W117_4* | W117_5* | W117_6* |
| --- | --- | --- | --- | --- | --- | --- | --- | --- | --- | --- | --- | --- | --- | --- |
| Rock | FQM | FQM | FQM | FQM | CQM | CQM | CQM | CQM | CQM | CQM | CQM | FQM | FQM | FQM |
| Depth(m) | 3679 | 3678.92 | 3639.63 | 3676.6 | 3634.08 | 3628.2 | 3629.28 | 3631.65 | 3627.35 | 3631.6 | 3639 | 3676 | 3678.26 | 3679 |
| SiO_2_ | 76.59 | 77.30 | 77.93 | 76.20 | 75.29 | 73.34 | 75.75 | 74.45 | 72.26 | 74.03 | 75.73 | 75.18 | 73.35 | 75.61 |
| TiO_2_ | 0.09 | 0.08 | 0.13 | 0.07 | 0.19 | 0.19 | 0.21 | 0.20 | 0.30 | 0.46 | 0.43 | 0.21 | 0.42 | 0.32 |
| Al_2_O_3_ | 12.52 | 12.15 | 11.03 | 12.21 | 12.85 | 13.01 | 12.51 | 12.91 | 13.07 | 12.19 | 11.21 | 12.25 | 11.95 | 11.43 |
| TFe_2_O_3_ | 2.03 | 1.89 | 2.07 | 1.88 | 2.40 | 1.56 | 3.00 | 2.29 | 1.70 | 2.55 | 3.00 | 2.09 | 2.89 | 1.91 |
| MnO | 0.02 | 0.02 | 0.01 | 0.02 | 0.02 | 0.03 | 0.02 | 0.01 | 0.02 | 0.03 | 0.03 | 0.03 | 0.04 | 0.03 |
| MgO | 0.23 | 0.16 | 0.26 | 0.17 | 0.21 | 0.82 | 0.29 | 0.31 | 0.58 | 0.74 | 0.52 | 0.40 | 0.63 | 0.45 |
| CaO | 0.31 | 0.38 | 0.96 | 0.45 | 0.19 | 1.06 | 0.16 | 0.36 | 1.03 | 0.67 | 0.67 | 0.60 | 1.50 | 1.16 |
| Na_2_O | 2.74 | 2.80 | 1.98 | 2.82 | 2.47 | 1.75 | 2.24 | 2.27 | 2.27 | 2.62 | 2.08 | 2.75 | 2.65 | 2.79 |
| K_2_O | 5.65 | 5.46 | 5.95 | 5.59 | 6.67 | 6.61 | 6.06 | 6.97 | 7.31 | 5.76 | 5.44 | 5.55 | 5.27 | 5.05 |
| P_2_O_5_ | 0.05 | 0.04 | 0.07 | 0.04 | 0.07 | 0.10 | 0.08 | 0.07 | 0.08 | 0.09 | 0.11 | 0.06 | 0.09 | 0.07 |
| LOI | 0.68 | 0.65 | 0.44 | 1.21 | 0.57 | 1.99 | 0.99 | 1.07 | 1.40 | 0.96 | 0.94 | 0.95 | 1.39 | 1.24 |
| Total alkali | 8.39 | 8.26 | 7.93 | 8.41 | 9.14 | 8.36 | 8.30 | 9.24 | 9.58 | 8.38 | 7.52 | 8.30 | 7.92 | 7.84 |
| Mg# | 20 | 15 | 22 | 17 | 16 | 53 | 17 | 23 | 43 | 39 | 28 | 30 | 32 | 34 |
| ACNK | 1.12 | 1.09 | 0.96 | 1.06 | 1.11 | 1.09 | 1.19 | 1.08 | 0.97 | 1.04 | 1.07 | 1.05 | 0.93 | 0.94 |
| Li | 25.5 | 20.7 | 24.2 | 23.1 | 19.7 | 25.7 | 32.3 | 26.5 | 16 | 13.2 | 28.2 | 21.5 | 16.8 | 16.2 |
| Sc | 3.66 | 3.35 | 2.07 | 3.33 | 3.95 | 6.01 | 4.59 | 4.31 | 3.4 | 4.3 | 5.66 | 4.8 | 6.43 | 4.49 |
| V | 4.71 | 3.17 | 5.69 | 2.68 | 8.84 | 12.5 | 8.79 | 9.5 | 30.6 | 42.7 | 32.6 | 19.5 | 43.5 | 31 |
| Cr | 2.87 | 1.8 | 1.45 | 5.8 | 2.83 | 2.49 | 3.17 | 1.82 | 131 | 166 | 145 | 163 | 152 | 98.5 |
| Co | 0.663 | 0.695 | 1.83 | 0.806 | 1.01 | 0.453 | 0.836 | 0.898 | 3.33 | 6.08 | 4.56 | 2.9 | 5.95 | 3.73 |
| Ni | 1.52 | 1.46 | 6.93 | 7.23 | 1.83 | 2.09 | 1.4 | 1.06 | 8.44 | 9.87 | 7.51 | 4.77 | 9.49 | 7.17 |
| Cu | 3.34 | 2.61 | 6.75 | 2.79 | 2.79 | 1.77 | 3.05 | 2.58 | 20.4 | 19.5 | 26.8 | 16 | 38.3 | 26.4 |
| Zn | 16.5 | 16.3 | 12.7 | 15.9 | 24.1 | 14.2 | 20.3 | 17.6 | 18 | 33.6 | 28.7 | 23.4 | 30.4 | 24.2 |
| Ga | 22.1 | 18.9 | 19.8 | 20.7 | 24.8 | 37 | 25.9 | 25.8 | 17.1 | 17.2 | 19.1 | 20.5 | 18.8 | 18.3 |
| Rb | 450 | 419 | 304 | 420 | 357 | 414 | 354 | 355 | 335 | 302 | 308 | 389 | 360 | 358 |
| Sr | 14 | 19.9 | 20.2 | 20.6 | 30.3 | 28.3 | 26.7 | 30.4 | 70.1 | 57.1 | 46.3 | 29.5 | 65 | 70.4 |
| Y | 102 | 92.4 | 61.8 | 119 | 50.2 | 88.4 | 54.2 | 64.7 | 28.9 | 40.9 | 65 | 75.8 | 96.9 | 95.1 |
| Zr | 91.3 | 87.7 | 26.6 | 91.8 | 160 | 198 | 216 | 191 | 102 | 142 | 176 | 174 | 178 | 169 |
| Nb | 13.2 | 12.2 | 6.35 | 11.5 | 11.7 | 12.7 | 12.8 | 12.5 | 8.07 | 9.52 | 15.6 | 12.6 | 14.7 | 11.6 |
| Cs | 22.5 | 19.3 | 13.6 | 20 | 12.7 | 19.3 | 15.4 | 15.7 | 10.7 | 10.8 | 16.2 | 14.6 | 12.3 | 16.1 |
| Ba | 46.9 | 35.3 | 203 | 39.4 | 405 | 232 | 303 | 357 | 328 | 380 | 194 | 75.5 | 80.8 | 82 |
| La | 29.6 | 22.5 | 39.8 | 26.9 | 50.1 | 81.6 | 53.7 | 54 | 32 | 31.9 | 46.7 | 24.2 | 32.8 | 69.1 |
| Ce | 68.7 | 53 | 81.5 | 65.2 | 113 | 178 | 117 | 116 | 60.7 | 66.2 | 100 | 53.8 | 69.9 | 156 |
| Pr | 8.85 | 6.76 | 10.4 | 8.15 | 13.2 | 22.1 | 13.9 | 14 | 6.66 | 7.25 | 11.7 | 6.66 | 8.54 | 20.1 |
| Nd | 33.2 | 25.1 | 38.9 | 30.6 | 48.8 | 81.8 | 52 | 51.6 | 25.4 | 29.3 | 42.8 | 25.2 | 32.9 | 74.9 |
| Sm | 9.48 | 7.28 | 8.96 | 9.19 | 10.6 | 18 | 11.3 | 11 | 4.62 | 6 | 8.9 | 6.49 | 8.57 | 18.7 |
| Eu | 0.243 | 0.149 | 0.372 | 0.137 | 0.479 | 0.684 | 0.469 | 0.501 | 0.691 | 0.804 | 0.506 | 0.281 | 0.428 | 0.528 |
| Gd | 10.3 | 8.78 | 8.45 | 10.8 | 8.97 | 15.4 | 9.47 | 9.34 | 4.89 | 5.58 | 8.01 | 6.82 | 9.44 | 15.7 |
| Tb | 2.46 | 2.3 | 1.88 | 2.87 | 1.68 | 2.88 | 1.79 | 1.95 | 1.04 | 1.24 | 1.8 | 1.9 | 2.41 | 2.93 |
| Dy | 16.5 | 16.1 | 10.9 | 19.4 | 9.7 | 17.1 | 9.89 | 12 | 5.66 | 7.54 | 12.2 | 12.4 | 16 | 16.2 |
| Ho | 3.26 | 3.26 | 2.1 | 4.14 | 1.8 | 3.25 | 1.97 | 2.39 | 1.03 | 1.51 | 2.26 | 2.73 | 3.43 | 3.4 |
| Er | 10.4 | 10.2 | 5.98 | 13.5 | 5.04 | 9.12 | 5.9 | 7.05 | 3.1 | 4.59 | 6.63 | 8.4 | 10.5 | 9.88 |
| Tm | 1.74 | 1.79 | 0.926 | 2.1 | 0.861 | 1.35 | 0.848 | 1.17 | 0.453 | 0.785 | 1.13 | 1.54 | 1.71 | 1.7 |
| Yb | 11 | 11.2 | 5.48 | 13.1 | 5.51 | 8.64 | 5.89 | 7.64 | 3.07 | 5.43 | 6.99 | 10 | 11.5 | 10.8 |
| Lu | 1.39 | 1.46 | 0.706 | 1.8 | 0.739 | 1.14 | 0.826 | 0.958 | 0.458 | 0.711 | 0.891 | 1.54 | 1.55 | 1.53 |
| Hf | 4.57 | 4.6 | 1.46 | 4.77 | 6.06 | 7.24 | 8.02 | 6.97 | 3.28 | 4.64 | 5.83 | 6.86 | 6.71 | 6.68 |
| Ta | 1.93 | 1.67 | 0.706 | 1.72 | 1.35 | 1.55 | 1.53 | 1.28 | 1.1 | 1.61 | 1.64 | 2.1 | 2.18 | 1.73 |
| Pb | 18.2 | 19.5 | 21.9 | 18.2 | 24.7 | 17.6 | 21.7 | 34.2 | 38 | 27.4 | 26.1 | 18.4 | 17.5 | 15.8 |
| Th | 34.3 | 30.8 | 27.1 | 32 | 33.9 | 39.8 | 40.2 | 39.6 | 21.8 | 26.9 | 49.1 | 35.6 | 32.2 | 29.2 |
| U | 6.15 | 7.59 | 2.23 | 15.5 | 2.27 | 10.4 | 3.22 | 3.81 | 3 | 2.68 | 3.79 | 10.7 | 12.3 | 7.77 |
| ΣREE | 207 | 170 | 216 | 208 | 270 | 441 | 285 | 290 | 150 | 169 | 251 | 162 | 210 | 401 |
| (La/Yb)_N_ | 1.82 | 1.36 | 4.91 | 1.39 | 6.14 | 6.38 | 6.16 | 4.78 | 7.04 | 3.97 | 4.51 | 1.64 | 1.93 | 4.32 |
| (Gd/Yb)_N_ | 0.76 | 0.64 | 1.25 | 0.67 | 1.32 | 1.44 | 1.30 | 0.99 | 1.29 | 0.83 | 0.93 | 0.55 | 0.67 | 1.18 |
| (Ho/Yb)_N_ | 0.86 | 0.85 | 1.12 | 0.92 | 0.95 | 1.10 | 0.97 | 0.91 | 0.98 | 0.81 | 0.94 | 0.80 | 0.87 | 0.92 |
| Eu/Eu* | 0.08 | 0.06 | 0.13 | 0.04 | 0.15 | 0.13 | 0.14 | 0.15 | 0.44 | 0.42 | 0.18 | 0.13 | 0.15 | 0.09 |
| 10000Ga/Al | 3.33 | 2.94 | 3.39 | 3.20 | 3.65 | 5.37 | 3.91 | 3.77 | 2.47 | 2.67 | 3.22 | 3.16 | 2.97 | 3.02 |
| Zr/Hf | 20.0 | 19.1 | 18.2 | 19.3 | 26.4 | 27.4 | 26.9 | 27.4 | 31.1 | 30.6 | 30.2 | 25.4 | 26.5 | 25.3 |
| T_Zr_(℃) | 751 | 745 | 650 | 746 | 795 | 812 | 831 | 808 | 742 | 778 | 803 | 799 | 787 | 786 |

**Note:** The samples with (*) are from Gu et al (2013)^48^. LOI=Loss on ignition; Mg#=Mg^2+^/(Mg^2+^+Fe^2+^)×100; Eu/Eu*=Eu_N_/(Sm_N_×Gd_N_)^1/2^;

A/NK=molar Al_2_O_3_/(Na_2_O+K_2_O); A/CNK=molar Al_2_O_3_/(Na_2_O+K_2_O+CaO); N=chondrite-normalized data

FQM = Fine-grained quartz monzonite; CQM = Coarse-grained quartz monzonite

**Neoproterozoic rifting in the Upper Yangtze Continental Block: Constraints from granites in the Well W117 borehole, South China**

Deng-Fa He*, Di Li*, Chuan-Xin Li, Ying-Qiang Li, Qing-Hua Mei

**Table S3 Whole–rock Sr–Nd isotopic compositions of the Well W117 quartz monzonites in the central Sichuan Basin.**

| Sample | Rock | Rb(ppm) | Sr(ppm) | ^87^Rb/^86^Sr | ^87^Sr/^86^Sr | 2σ | (^87^Sr/^86^Sr)_i_ | Sm(ppm) | Nd(ppm) | ^147^Sm/^144^Nd | ^143^Nd/^144^Nd | 2σ | ε_Nd_(t) | T_2DM_(Ga) |
| --- | --- | --- | --- | --- | --- | --- | --- | --- | --- | --- | --- | --- | --- | --- |
| 117-1 | FQM | 450 | 14.0 | 102.240 | 1.723621 | 31 | 1.330882 | 9.48 | 33.2 | 0.1726 | 0.512227 | 6 | -5.5 | 1.76 |
| 117-2 | FQM | 419 | 19.9 | 65.012 | 1.394649 | 17 | 1.144915 | 7.28 | 25.1 | 0.1753 | 0.512210 | 6 | -6.1 | 1.80 |
| 117-3 | FQM | 304 | 20.2 | 45.507 | 1.169092 | 14 | 0.994282 | 8.96 | 38.9 | 0.1392 | 0.512062 | 8 | -5.3 | 1.75 |
| 117-4 | CQM | 357 | 30.3 | 35.330 | 1.079794 | 13 | 0.944080 | 10.6 | 48.8 | 0.1313 | 0.511994 | 6 | -5.8 | 1.78 |
| 117-5 | CQM | 414 | 28.3 | 44.054 | 1.125333 | 12 | 0.956104 | 18.0 | 81.8 | 0.1330 | 0.511996 | 7 | -5.9 | 1.79 |
| 117-20 | CQM | 354 | 26.7 | 39.964 | 1.135100 | 12 | 0.981585 | 11.3 | 52.0 | 0.1314 | 0.511996 | 7 | -5.7 | 1.78 |
| 117-39 | FQM | 420 | 20.6 | 62.920 | 1.389073 | 14 | 1.147373 | 9.19 | 30.6 | 0.1815 | 0.512197 | 7 | -7.0 | 1.87 |
| 117-40 | CQM | 355 | 30.4 | 35.050 | 1.090034 | 15 | 0.955395 | 11.0 | 51.6 | 0.1289 | 0.511998 | 7 | -5.4 | 1.76 |

**Note:** *T*_2DM_=(1/λ)ln(1+((^143^Nd/^144^Nd)_S_−(^143^Nd/^144^Nd)_DM_−((^147^Sm/^144^Nd)_S_−(^147^Sm/^144^Nd)_C_(e^λt^−1))/((^147^Sm/^144^Nd)_C_−(^147^Sm/^144^Nd)_DM_)).

FQM = Fine-grained quartz monzonite; CQM = Coarse-grained quartz monzonite

**Neoproterozoic rifting in the Upper Yangtze Continental Block: Constraints from granites in the Well W117 borehole, South China**

Deng-Fa He*, Di Li*, Chuan-Xin Li, Ying-Qiang Li, Qing-Hua Mei

**Table S4 Results of Lu–Hf isotopic analysis of zircons from the Well W117 quartz monzonites in the central Sichuan Basin.**

| Spots | Age(Ma) | ^176^Yb/^177^Hf | ^176^Lu/^177^Hf | ^176^Hf/^177^Hf | ±2σ | *ε*_Hf_(t) | T_1DM_(Ga) | T_2DM_(Ga) | *f*_Lu/Hf_ |
| --- | --- | --- | --- | --- | --- | --- | --- | --- | --- |
| Sample 117-3 |  |  |  |  |  |  |  |  |  |
| #03 | 829.8 | 0.039052 | 0.001544 | 0.282230 | 0.000025 | -1.9 | 1.46 | 1.84 | -0.95 |
| #04 | 829.8 | 0.028147 | 0.001108 | 0.282273 | 0.000021 | -0.2 | 1.39 | 1.73 | -0.97 |
| #05 | 829.8 | 0.035255 | 0.001353 | 0.282193 | 0.000033 | -3.1 | 1.51 | 1.92 | -0.96 |
| #06 | 829.8 | 0.036348 | 0.001254 | 0.282162 | 0.000037 | -4.2 | 1.55 | 1.98 | -0.96 |
| #07 | 829.8 | 0.049695 | 0.001714 | 0.282154 | 0.000061 | -4.7 | 1.58 | 2.02 | -0.95 |
| #08 | 829.8 | 0.050983 | 0.001885 | 0.282010 | 0.000066 | -9.9 | 1.79 | 2.34 | -0.94 |
| #09 | 829.8 | 0.050725 | 0.001603 | 0.281894 | 0.000086 | -13.9 | 1.94 | 2.59 | -0.95 |
| #11 | 829.8 | 0.041902 | 0.001627 | 0.282045 | 0.000033 | -8.5 | 1.73 | 2.25 | -0.95 |
| #12 | 829.8 | 0.029056 | 0.001099 | 0.282111 | 0.000034 | -5.9 | 1.61 | 2.09 | -0.97 |
| #13 | 829.8 | 0.042337 | 0.001527 | 0.282099 | 0.000036 | -6.6 | 1.65 | 2.13 | -0.95 |
| #14 | 829.8 | 0.039593 | 0.001396 | 0.282279 | 0.000027 | -0.1 | 1.39 | 1.72 | -0.96 |
| #16 | 829.8 | 0.029889 | 0.001163 | 0.282185 | 0.000028 | -3.3 | 1.51 | 1.93 | -0.96 |
| #17 | 829.8 | 0.040210 | 0.001572 | 0.282303 | 0.000018 | 0.6 | 1.36 | 1.68 | -0.95 |
| #18 | 829.8 | 0.032262 | 0.001224 | 0.282065 | 0.000033 | -7.6 | 1.68 | 2.20 | -0.96 |
| #19 | 829.8 | 0.040624 | 0.001366 | 0.282194 | 0.000034 | -3.1 | 1.51 | 1.91 | -0.96 |
| #20 | 829.8 | 0.036997 | 0.001387 | 0.282168 | 0.000035 | -4.0 | 1.54 | 1.97 | -0.96 |
| #21 | 829.8 | 0.036092 | 0.001417 | 0.282237 | 0.000031 | -1.6 | 1.45 | 1.82 | -0.96 |
| #22 | 829.8 | 0.028823 | 0.001037 | 0.281991 | 0.000037 | -10.1 | 1.78 | 2.36 | -0.97 |
| #23 | 829.8 | 0.046731 | 0.001798 | 0.282341 | 0.000022 | 1.8 | 1.32 | 1.60 | -0.95 |
| #24 | 829.8 | 0.035814 | 0.001406 | 0.282339 | 0.000029 | 2.0 | 1.30 | 1.59 | -0.96 |
| #25 | 829.8 | 0.022815 | 0.000890 | 0.282217 | 0.000021 | -2.0 | 1.46 | 1.84 | -0.97 |
| #26 | 829.8 | 0.044525 | 0.001674 | 0.282155 | 0.000033 | -4.7 | 1.57 | 2.01 | -0.95 |
| #27 | 829.8 | 0.027612 | 0.001098 | 0.282284 | 0.000024 | 0.2 | 1.37 | 1.70 | -0.97 |
| #28 | 829.8 | 0.035726 | 0.001356 | 0.282196 | 0.000025 | -3.0 | 1.50 | 1.91 | -0.96 |
| Sample 117-4 |  |  |  |  |  |  |  |  |  |
| #01 | 813.8 | 0.053098 | 0.001993 | 0.282240 | 0.000026 | -2.2 | 1.47 | 1.84 | -0.94 |
| #02 | 813.8 | 0.034419 | 0.001330 | 0.282243 | 0.000023 | -1.7 | 1.44 | 1.81 | -0.96 |
| #03 | 813.8 | 0.032240 | 0.001236 | 0.282218 | 0.000026 | -2.5 | 1.47 | 1.86 | -0.96 |
| #04 | 813.8 | 0.038708 | 0.001405 | 0.282252 | 0.000023 | -1.4 | 1.43 | 1.79 | -0.96 |
| #06 | 813.8 | 0.064511 | 0.002157 | 0.282184 | 0.000028 | -4.2 | 1.55 | 1.97 | -0.94 |
| #08 | 813.8 | 0.042330 | 0.001450 | 0.282167 | 0.000037 | -4.5 | 1.55 | 1.99 | -0.96 |
| #09 | 813.8 | 0.032199 | 0.001243 | 0.282324 | 0.000029 | 1.2 | 1.32 | 1.62 | -0.96 |
| #10 | 813.8 | 0.034251 | 0.001302 | 0.281943 | 0.000042 | -12.3 | 1.86 | 2.48 | -0.96 |
| #11 | 813.8 | 0.053934 | 0.002008 | 0.282253 | 0.000035 | -1.7 | 1.45 | 1.81 | -0.94 |
| #12 | 813.8 | 0.049988 | 0.001900 | 0.282301 | 0.000020 | 0.0 | 1.38 | 1.70 | -0.94 |
| #13 | 813.8 | 0.028029 | 0.001110 | 0.282270 | 0.000015 | -0.6 | 1.39 | 1.74 | -0.97 |
| #14 | 813.8 | 0.037078 | 0.001377 | 0.282292 | 0.000021 | 0.0 | 1.37 | 1.70 | -0.96 |
| Sample 117-5 |  |  |  |  |  |  |  |  |  |
| #01 | 813.8 | 0.045528 | 0.001694 | 0.282253 | 0.000030 | -1.5 | 1.44 | 1.80 | -0.95 |
| #02 | 813.8 | 0.039265 | 0.001522 | 0.282314 | 0.000018 | 0.7 | 1.34 | 1.66 | -0.95 |
| #03 | 813.8 | 0.066543 | 0.002436 | 0.282360 | 0.000033 | 1.8 | 1.31 | 1.59 | -0.93 |
| #04 | 813.8 | 0.056994 | 0.002285 | 0.282276 | 0.000034 | -1.1 | 1.43 | 1.77 | -0.93 |
| #05 | 813.8 | 0.043626 | 0.001703 | 0.282271 | 0.000032 | -0.9 | 1.41 | 1.76 | -0.95 |
| #06 | 813.8 | 0.024772 | 0.000969 | 0.282230 | 0.000034 | -2.0 | 1.44 | 1.83 | -0.97 |
| #07 | 813.8 | 0.038595 | 0.001472 | 0.282279 | 0.000032 | -0.5 | 1.39 | 1.74 | -0.96 |
| #08 | 813.8 | 0.035090 | 0.001399 | 0.282316 | 0.000026 | 0.8 | 1.34 | 1.65 | -0.96 |
| #09 | 813.8 | 0.055423 | 0.002204 | 0.282267 | 0.000028 | -1.3 | 1.44 | 1.79 | -0.93 |
| #10 | 813.8 | 0.042576 | 0.001522 | 0.282206 | 0.000026 | -3.1 | 1.50 | 1.90 | -0.95 |
| #11 | 813.8 | 0.053614 | 0.001965 | 0.282136 | 0.000031 | -5.8 | 1.61 | 2.07 | -0.94 |
| #12 | 813.8 | 0.042006 | 0.001684 | 0.282306 | 0.000020 | 0.3 | 1.36 | 1.68 | -0.95 |
| #13 | 813.8 | 0.090013 | 0.003211 | 0.282240 | 0.000034 | -2.8 | 1.52 | 1.88 | -0.90 |
| #14 | 813.8 | 0.054599 | 0.002083 | 0.282368 | 0.000020 | 2.3 | 1.29 | 1.55 | -0.94 |
| #15 | 813.8 | 0.033705 | 0.001297 | 0.282200 | 0.000027 | -3.2 | 1.50 | 1.91 | -0.96 |
| #16 | 813.8 | 0.056356 | 0.002125 | 0.282298 | 0.000021 | -0.2 | 1.39 | 1.72 | -0.94 |
| #17 | 813.8 | 0.033098 | 0.001282 | 0.282252 | 0.000019 | -1.3 | 1.42 | 1.79 | -0.96 |
| #18 | 813.8 | 0.059235 | 0.002148 | 0.282085 | 0.000041 | -7.7 | 1.70 | 2.19 | -0.94 |
| #19 | 813.8 | 0.041265 | 0.001594 | 0.282099 | 0.000031 | -7.0 | 1.65 | 2.14 | -0.95 |
| #20 | 813.8 | 0.027110 | 0.001091 | 0.282318 | 0.000021 | 1.1 | 1.32 | 1.63 | -0.97 |
| #21 | 813.8 | 0.029658 | 0.001174 | 0.282323 | 0.000022 | 1.2 | 1.32 | 1.62 | -0.96 |
| #22 | 813.8 | 0.046750 | 0.001732 | 0.282266 | 0.000022 | -1.1 | 1.42 | 1.77 | -0.95 |

**Note:** ^176^Lu decay constant is 1.865×10^−11^ yr^−1^; Chondritic values: ^176^Lu/^177^Hf =0.0332±0. 0002, ^176^Hf/^177^Hf =0. 282772±0.000029;

Depleted mantle values: (^176^Lu/^177^Hf)_DM_=0.0384, (^176^Hf/^177^Hf)_DM_=0.28325;

*T*_DM1_=1/λ×ln(1+((^176^Hf/^177^Hf)_S_−(^176^Hf/^177^Hf)_DM_)/((^176^Lu/^177^Hf)_S_−(^176^Lu/^177^Hf)_DM_));

*T*_DM2_=1/λ×ln(1+((^176^Hf/^177^Hf)_S,t_−(^176^Hf/^177^Hf)_DM,t_)/((^176^Lu/^177^Hf)_S_−(^176^Lu/^177^Hf)_DM_))+t
